# Supplementary material for: Improved alpharetrovirus-based Gag.MS2 particles for efficient and transient delivery of CRISPR-Cas9 into target cells
Source: Mol Ther Nucleic Acids. 2022 Jan 1;27:810–23. doi: 10.1016/j.omtn.2021.12.033 (PMC8801357; doi:10.1016/j.omtn.2021.12.033)
Supplement: Document S1. Figures S1–S5 and Table S1 [file mmc1.pdf]

OMTN, Volume *27*

Supplemental information

Improved alpharetrovirus-based Gag.MS2 particles for efficient and transient delivery of CRISPR-Cas9 into target cells

Yvonne Baron, Johanna Sens, Lucas Lange, Larissa Nassauer, Denise Klatt, Dirk Hoffmann, Marc-Jens Kleppa, Philippe Vollmer Barbosa, Maximilian Keisker, Viviane Steinberg, Julia D. Suerth, Florian W.R. Vondran, Johann Meyer, Michael Morgan, Axel Schambach, and Melanie Galla

Supplemental Material and Methods

Cloning strategies

Lentiviral CRISPR/Cas9 all-in-one vector plasmids pLKO5.hU6.sgRNA.Tet2.PPT.EFS.SpCas9.P2A. EGFP.gPRE (HIV-1-based LIT.CRISPR.Tet2)[1] as well as the LIT.SFFV.RFP657.Tet2 reporter construct (pLKO5.PPT.SF.RFP657.Tet2.i2.Puro.PRE)[1] were designed and generated in former studies.

To enable silencing resistant expression of the *RFP657.Tet2* reporter cassette in hiPSC, we replaced the SFFV promoter in the LIT.SFFV.RFP657.Tet2 reporter depicted in Figure S1A[1] by a promoter configuration which was validated to most efficiently inhibit epigenetic silencing in hiPSC.[2] The promoter configuration consisted of the minimal ubiquitous chromatin opening element CBX3[3] juxtaposed to the elongation factor 1 alpha short (EFS) promoter and was inserted as an XhoI/AgeI fragment into the LIT.SFFV.RFP657.Tet2 reporter plasmid. The resulting lentiviral reporter plasmid was named LIT.CBX.EFS.RFP657.Tet2 (pLKO5.PPT.CBX3.EFS.RFP657.Tet2.i2.Puro.PRE).

The cloning strategies for g.CA.pr.MS2CP (pcDNA3.MLV.Gag.CA.pr.MS2CP), g.Pol, *SpCas9.TS*, and Tet2.TS, TP53.TS, Trp53.TS or CXCR4.TS sgRNA expression constructs are described elsewhere.[4]

For EGFP.TS.inc sgRNA expression a non-retroviral expression plasmid was generated (pCMV.DsRedexp.hU6.sgRNA.EGFP2.TS.inc.pA) by introducing phosphorylated and annealed oligonucleotides 5'-CACCGCACTGCACGCCGTAGGTCA-3' and 5'-AAACTGACCTACGGCGTGCA GTGC-3' into the BsmBI site of pCMV.DsRedexp.hU6.BsmBI-Stuffer.TS.inc.[4]

For the generation of pcDNA3.ASLV.Gag.co.NC.pr.MS2 (a.Gag.MS2), we first generated alpharetroviral Gag expression plasmids with codon-optimized subdomains (MA, p2, p10, CA and NC) with or without the retroviral protease site adjacent to NC. The codon-optimized incomplete a.Gag fragment lacking the PR subdomain was amplified with primers 5'-CGTGAGATCTGAATTCGC CACCATG-3' and 5'-CCATACCGGTGCTCACGGCCAGGCTCACGGCAGGCTC-3' from the codon-optimized alpharetroviral wt Gag-Pol expression plasmid (pcDNA3.wtAlpha.G/P.co).[5] Subsequently, the PCR fragments were inserted into the EcoRI/AgeI digested pcDNA3.ASLV.Gag.NC-GFP backbone (Gag part is not codon-optimized) to generate codon-optimized (Gag part only) pcDNA3.ASLV.Gag.co.NC.pr.EGFP. Finally, the *EGFP* transgene was replaced by the *MS2CP* heterodimer sequence[4] by restriction enzymes AgeI and NotI.

Genetic detection of HDR events

For genetic analysis of a.Gag.MS2.CRISPR.HDR-induced HDR events in HT1080-based EGFP reporter cells, selected cell samples (treated or untreated) from Figure 6B were sorted for EBFP positive cells 13 d after transduction. gDNA was isolated from these cell populations with the QIAamp DNA Blood Mini Kit (QIAGEN) following the manufacturer's instructions. Subsequently, the respective locus of the *EGFP/EBFP* gene was amplified by primers 5'-ATGGTGAGCAAGGGCGAGGAG-3' and 5'-CTTGTAC AGCTCGTCCATGCCG-3' using the Phire Tissue Direct PCR Master Mix (Thermo Fisher Scientific). Finally, the obtained PCR fragment was sequenced with the primer 5'-TTGAAGTTCACCTTGATG CCG-3' and the sequencing results were analyzed by the online ICE v2 CRISPR Analysis Tool (Synthego).[6] For the molecular conformation of HDR events in NuFF-based

EGFP reporter cells, selected bulk cultures from Figure 6C were harvested 3 d after transduction according to the instructions of the Thermo Scientific Phire Tissue Direct PCR Master Mix kit. The *EGFP/EBFP* locus was amplified as described above and the resulting amplicons were subcloned into a shuttle vector. After transformation into XL1-blue *Escherichia coli* bacteria (Agilent, Waldbronn, Germany), the plasmid DNA of bacterial colonies was extracted and analyzed for positive HDR events via sequencing and the online ICE v2 CRISPR Analysis Tool.

Table S1

Table S1: Comparison of Gag.MS2 particles and non-viral RNA delivery methods: Advantages and disadvantages of respective technologies.

RNA Delivery Method	Advantage(s)	Disadvantage(s)	Exemplary References
Naked RNA delivery	Simple; *in vivo* application; easy upscaling	Ineffective cellular uptake; prone to nuclease degradation; can be immunogenic; fast systemic clearance	[7–12]
Electroporation	Highly efficient; high reproducibility; easy upscaling	Voltage-induced cytotoxicity; no cell targeting; mainly for *ex-vivo* applications	[12–18]
Lipid-based nanocarriers	Moderate efficiency; low to moderate cytotoxicity; protection from nucleases; *in vivo* application; low immunogenicity	Inefficient endosomal escape; concerns about *in vivo* biodegradability; specific tissue targeting is challenging; cell type- and composition-dependent cytotoxicity; limitations in fabrication reproducibility	[12,17,19–23]
Polymer-based nanocarriers	Moderate efficiency; low to moderate cytotoxicity; protection from nuclease; *in vivo* application; capacity for cell-specific targeting; low immunogenicity; facile synthesis	Inefficient endosomal escape; cell type- and composition-dependent cytotoxicity	[12,17,20,23–26]
Direct Ligand-RNA conjugates	Limited cytotoxicity; *in vivo* application; cell-specific targeting	High RNA doses required; dependent on RNA-stabilizing modifications; only shown for small regulatory RNA	[11,27–30]
Retrovirus-based Gag.MS2 particles	Highly efficient; delivery of non-retroviral RNA; low immunogenicity; efficient endosomal escape; capacity for cell-specific targeting and *in vivo* application; relatively well-tolerated procedure	Efficiency is dependent on cellular co-factor and/or antiviral restriction factor expression; producer cells needed for particle generation	[4,31–40]<br>this paper

Figure S1

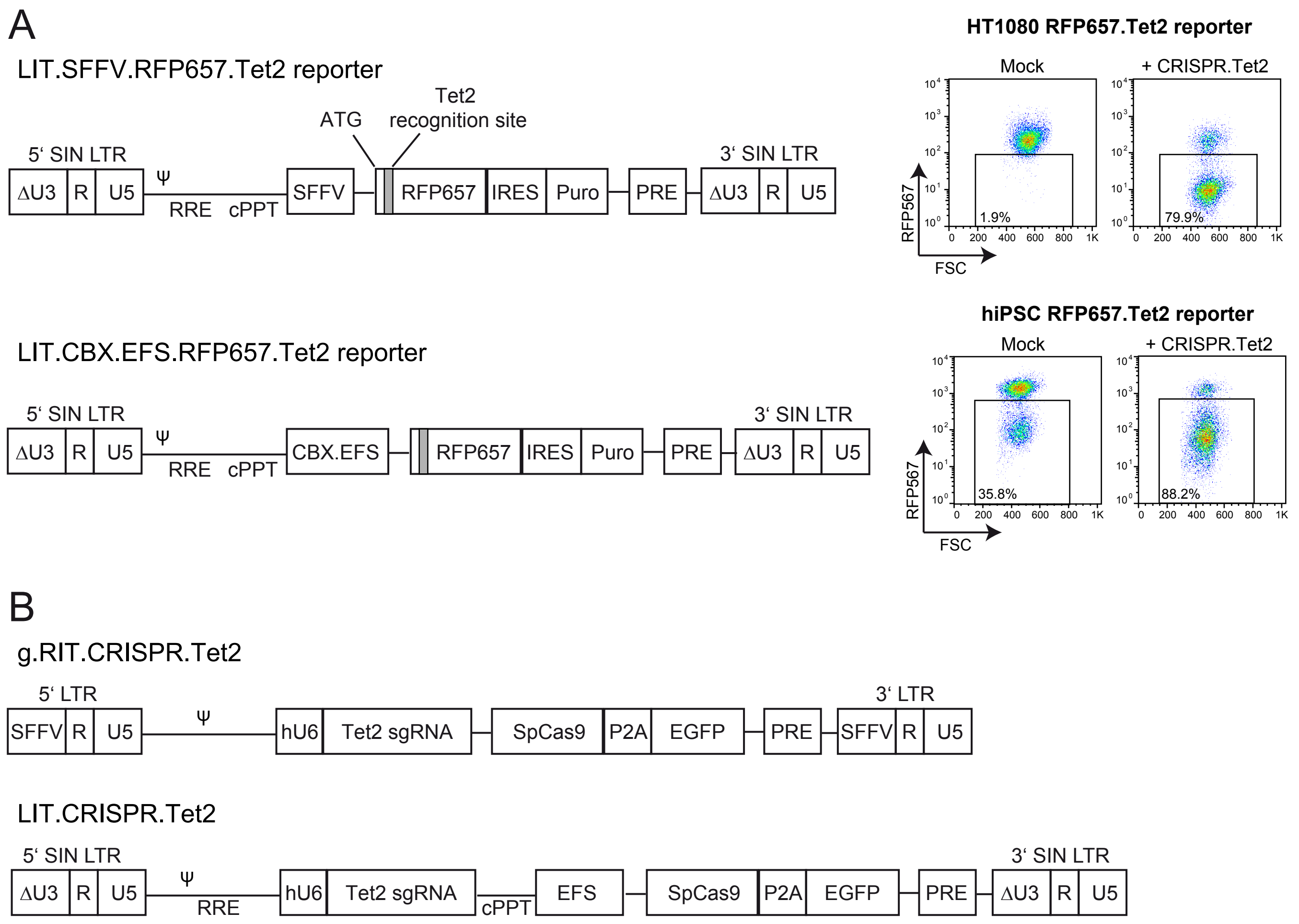

Figure S1. Schematic illustration of integrating retroviral vectors used for CRISPR/Cas9 experiments in this study. (**A**) Lentiviral integrating transfer (LIT) vector encoding the RFP657.Tet2 reporter cassette. The far-red *RFP657* gene is expressed from the spleen focus forming virus (SFFV) promoter (top) or the silencing resistant CBX3.EFS promoter (bottom). The latter consists of a minimal ubiquitous chromatin opening element (CBX3) fused to the elongation factor 1 alpha short (EFS) promoter. The *RFP657* gene contains the sgRNA recognition site (including native AGG PAM) for the mouse *Tet2* gene downstream of its start codon (ATG). *RFP657.Tet2* is expressed together with the puromycin resistance gene (Puro) via the internal ribosomal entry site (IRES) of the endomyocarditis virus. Representative FACS plots of non-transduced (Mock) or a.Gag.MS2.CRISPR.Tet2-transduced HT1080- or hiPSC-based RFP657.Tet2 reporters are shown on the right. (**B**) LIT and gammaretroviral transfer (g.RIT) CRISPR/Cas9 all-in-one vectors targeting mouse *Tet2*. In contrast to the long terminal repeat (LTR)-driven g.RIT.CRISPR.Tet2 vector, respective LIT vectors display self-inactivating (SIN) design. sgRNA expression is driven by the human Pol III promoter U6 (hU6), and the *Streptococcus pyogenes* Cas9 (SpCas9) is either co-expressed with green-fluorescent EGFP or the red-fluorescent dTomato protein via 2A self-cleavage sites from porcine teschovirus-1 (P2A) or thosea asigna virus (T2A). PRE: post-transcriptional regulatory element from woodchuck hepatitis virus; Ψ: retroviral packaging signal; RRE: Rev responsive element; cPPT: central polypurine tract.

Figure S2

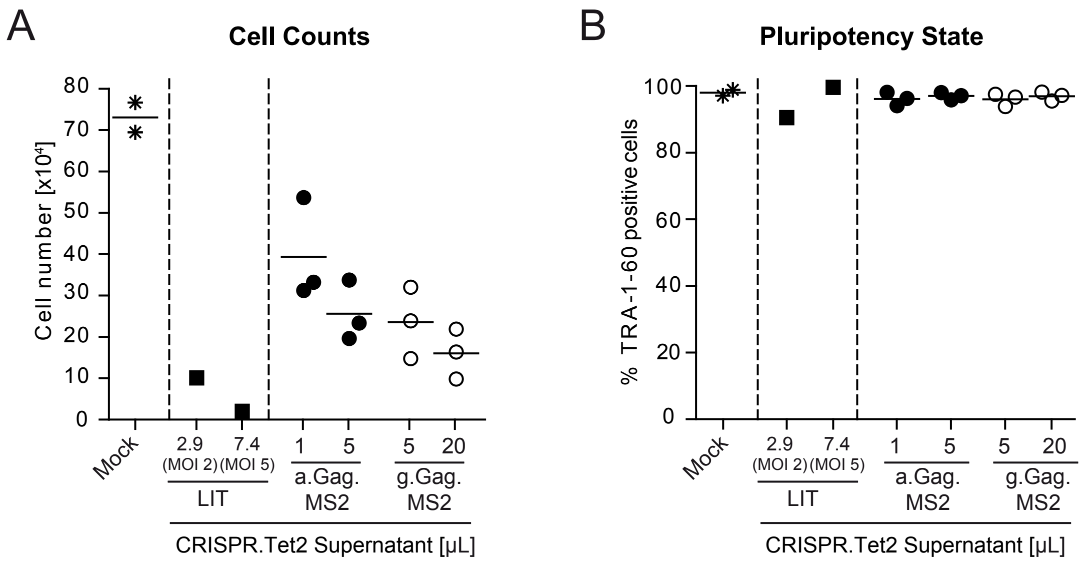

Figure S2. Reduced cytotoxicity in hiPSC that were treated with Gag.MS2-based CRISPR.Tet2 particles. Absolute cell numbers (**A**) and the respective pluripotency state (**B**) of transduced hiPSC-based RFP657.Tet2 reporter cultures from Figure 2D are shown. Integrating LIT.CRISPR.Tet2 particles served as control. Each data point represents one individually generated supernatant. TRA-1-60: pluripotency marker for hiPSC.

Figure S3

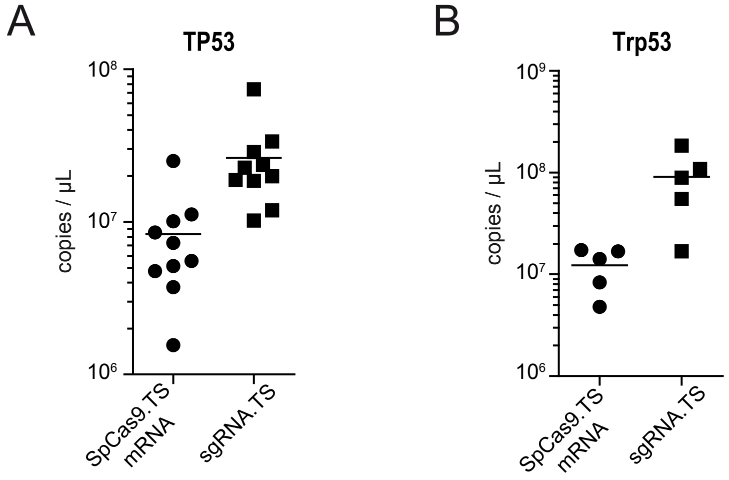

Figure S3. Quantification of SpCas9.TS mRNA and sgRNA.TS transcripts in a.Gag.MS2.CRISPR supernatants that were used for the transduction of primary cells (Figure 5). The concentrations of CRISPR RNA transcripts in supernatants containing particles targeting either human *TP53* (**A**) or murine *Trp53* (**B**) genes are shown.

Figure S4

A

Single-Stranded HDR Donor DNA Oligonucleotide (ssODN)

CCTTCGGGCATGGCGGACTTGAAGAAGTCGTGCTGCTTCATGTGGTCTGGGTAGCGGCTG
AAGCACTGCACGCCGTGGCTCAGTGTGGTCACGAGGGTGGGCCAGGGCACGGGCAGCTTG

B

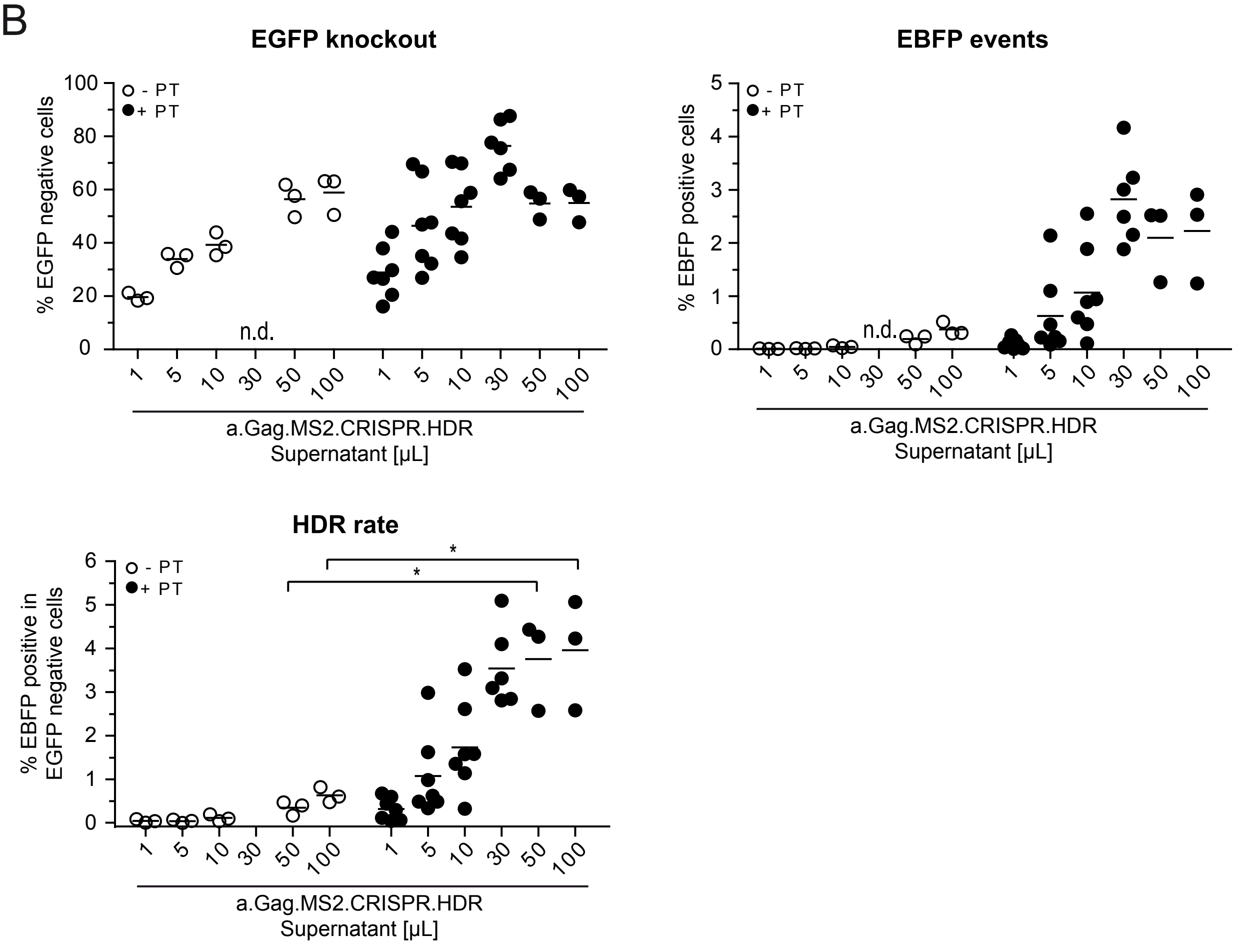

Figure S4. Efficient CRISPR/Cas9-induced HDR by a.Gag.MS2.CRISPR.HDR particles. Proof of concept was shown with the help of the recently published EGFP to EBFP transition model.[41] (**A**) Respective single-stranded DNA oligonucleotide (ssODN) donor sequence. The 120 bp long ssODN used in B, Figure 6 and Figure S5 contains two point mutations (in red) conferring the amino acid changes T66S and Y67H thereby switching EGFP to EBFP expression. The mutations are flanked by asymmetric homology arms (in black). An additional silent point mutation (in blue) within PAM should avoid CRISPR/Cas9-mediated cleavage of the HDR donor template. The black line indicates the EGFP.TS sgRNA target sequence with the exception of the 2 point mutations in red. The donor was purchased with and without phosphorothioate (+PT or -PT, respectively) modifications. (**B**) Co-transfected +PT ssODN during particle production enhanced HDR. +PT or -PT ssODN donors (0.43 nmol) were co-transfected during a.Gag.MS2.CRISPR.HDR particle production. HT1080-based EGFP HDR reporter cells were transduced with indicated supernatant volumes and were analyzed for EGFP$^-$ (upper left graph) and EBFP$^+$ positive cells (upper right graph) via flow cytometry. The calculated HDR rate (%EBFP$^+$ within EGFP$^-$ cells) is depicted at the lower left. The results of three to seven independent supernatants are depicted (N=3-7). n.d.: not determined.

Figure S5

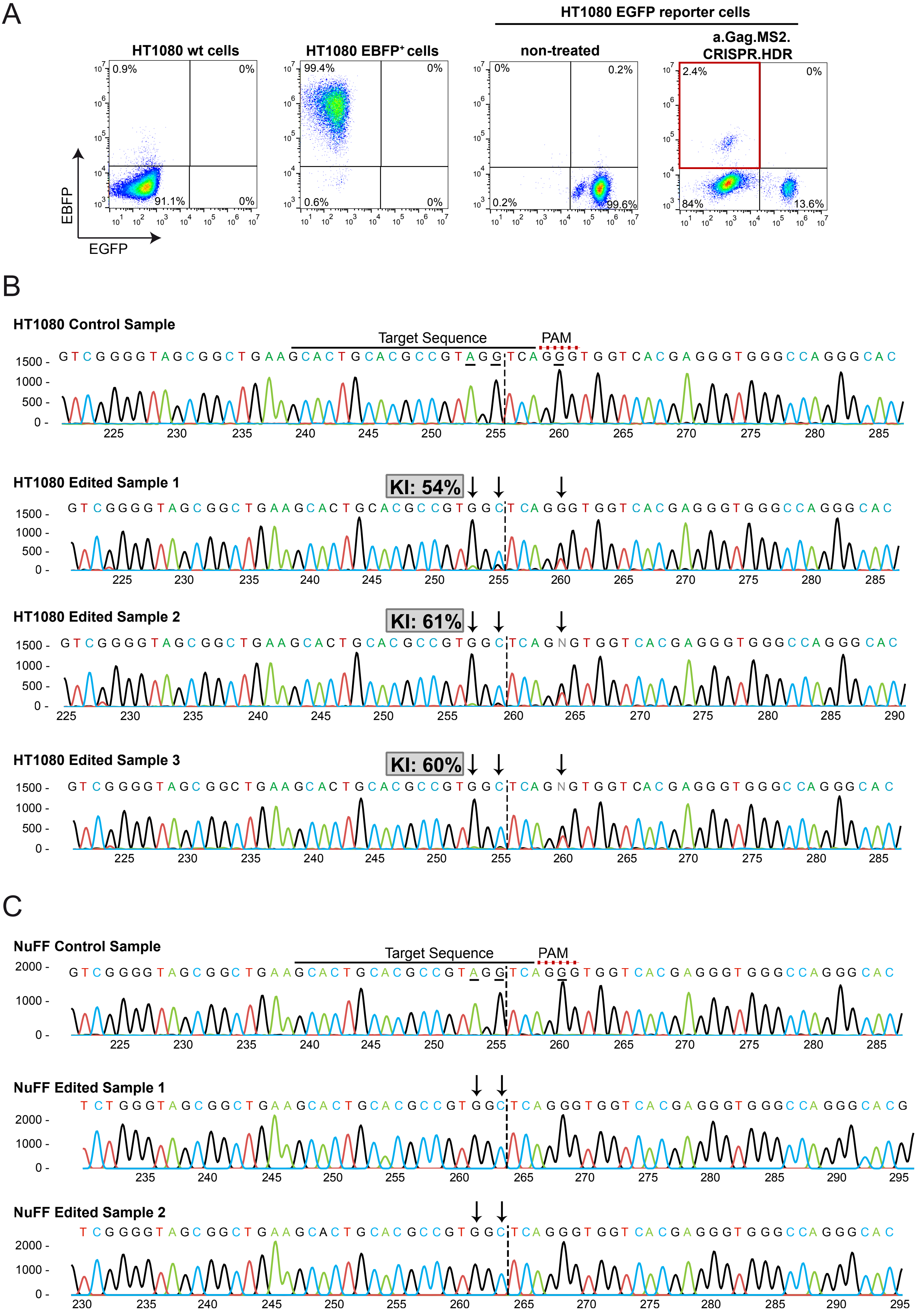
A
HT1080 EGFP reporter cells
HT1080 wt cells
HT1080 EBFP⁺ cells
non-treated
a.Gag.MS2.
CRISPR.HDR
0.9%
0%
91.1%
0%
99.4%
0%
0.6%
0%
0%
0.2%
0.2%
99.6%
2.4%
0%
84%
13.6%
EBFP
EGFP
B
HT1080 Control Sample
Target Sequence
PAM
GTCGGGGTAGCGGCTGAAGCACTGCACGCCGTAGGTCAGGGTGGTCACGAGGGTGGGCCAGGGCAC
HT1080 Edited Sample 1
KI: 54%
GTCGGGGTAGCGGCTGAAGCACTGCACGCCGTGGCTCAGGGTGGTCACGAGGGTGGGCCAGGGCAC
HT1080 Edited Sample 2
KI: 61%
GTCGGGGTAGCGGCTGAAGCACTGCACGCCGTGGCTCAGNGTGGTCACGAGGGTGGGCCAGGGCAC
HT1080 Edited Sample 3
KI: 60%
GTCGGGGTAGCGGCTGAAGCACTGCACGCCGTGGCTCAGNGTGGTCACGAGGGTGGGCCAGGGCAC
C
NuFF Control Sample
Target Sequence
PAM
GTCGGGGTAGCGGCTGAAGCACTGCACGCCGTAGGTCAGGGTGGTCACGAGGGTGGGCCAGGGCAC
NuFF Edited Sample 1
TCTGGGTAGCGGCTGAAGCACTGCACGCCGTGGCTCAGGGTGGTCACGAGGGTGGGCCAGGGCACG
NuFF Edited Sample 2
TCGGGGTAGCGGCTGAAGCACTGCACGCCGTGGCTCAGGGTGGTCACGAGGGTGGGCCAGGGCAC

Figure S5. Molecular analysis of HDR events in HT1080-based and NuFF-based EGFP reporter cells. (**A**) Representative flow cytometry plots showing the gating strategy for the sorting of edited $EBFP^+/EGFP^-$ cells (red box) 13 d after a.Gag.MS2.CRISPR.HDR treatment. (**B**) Knockin (KI) rate analyses of sorted $EBFP^+/EGFP^-$ cells. The genomic DNA of sorted cells from 3 independent experiments (Figure 6B) was extracted and sequenced. Resulting sequences were subjected to the ICE analysis tool (Synthego).[6] The „Edited Sample 1" is derived from cultures that were transduced without inhibitor. „Edited Samples 2 & 3" are derived from cells that were treated with a.Gag.MS2.CRISPR.HDR particles in the presence of the NHEJ inhibitor M3814. (**C**) Molecular analysis of HDR events in a.Gag.MS2.CRISPR.HDR-treated NuFF.EGFP reporter cells from Figure 6C. The respective site within *EGFP* was amplified and the amplicon was subcloned into a shuttle vector. After transformation, bacterial clones were screened for positive HDR events. Sequencing results were analyzed with the help of the ICE analysis tool. The switch from EGFP to EBFP in bulk cultures of two individual transductions (Edited Sample 1 and 2) is shown.

REFERENCES

1. Heckl, D., Kowalczyk, M.S., Yudovich, D., Belizaire, R., Puram, R. V, McConkey, M.E., Thielke, A., Aster, J.C., Regev, A., and Ebert, B.L. (2014). Generation of mouse models of myeloid malignancy with combinatorial genetic lesions using CRISPR-Cas9 genome editing. Nat. Biotechnol. *32*, 941–946.

2. Hoffmann, D., Schott, J.W., Geis, F.K., Lange, L., Müller, F.-J., Lenz, D., Zychlinski, D., Steinemann, D., Morgan, M., Moritz, T., et al. (2017). Detailed comparison of retroviral vectors and promoter configurations for stable and high transgene expression in human induced pluripotent stem cells. Gene Ther. *24*, 298–307.

3. Müller-Kuller, U., Ackermann, M., Kolodziej, S., Brendel, C., Fritsch, J., Lachmann, N., Kunkel, H., Lausen, J., Schambach, A., Moritz, T., et al. (2015). A minimal ubiquitous chromatin opening element (UCOE) effectively prevents silencing of juxtaposed heterologous promoters by epigenetic remodeling in multipotent and pluripotent stem cells. Nucleic Acids Res. *43*, 1577–1592.

4. Knopp, Y., Geis, F.K., Heckl, D., Horn, S., Neumann, T., Kuehle, J., Meyer, J., Fehse, B., Baum, C., Morgan, M., et al. (2018). Transient Retrovirus-Based CRISPR/Cas9 All-in-One Particles for Efficient, Targeted Gene Knockout. Mol. Ther. Nucleic Acids *13*, 256–274.

5. Suerth, J.D., Maetzig, T., Galla, M., Baum, C., and Schambach, A. (2010). Self-inactivating alpharetroviral vectors with a split-packaging design. J. Virol. *84*, 6626–6635.

6. Synthego (2019). Synthego Performance Analysis, ICE Analysis. v2.0. [accessed 2021/02/01].

7. Houseley, J., and Tollervey, D. (2009). The Many Pathways of RNA Degradation. Cell *136*, 763–776.

8. Tsui, N.B.Y., Ng, E.K.O., and Lo, Y.M.D. (2002). Stability of endogenous and added RNA in blood specimens, serum, and plasma. Clin. Chem. *48*, 1647–1653.

9. Wojtczak, B.A., Sikorski, P.J., Fac-Dabrowska, K., Nowicka, A., Warminski, M., Kubacka, D., Nowak, E., Nowotny, M., Kowalska, J., and Jemielity, J. (2018). 5′-Phosphorothiolate Dinucleotide Cap Analogues: Reagents for Messenger RNA Modification and Potent Small-Molecular Inhibitors of Decapping Enzymes. J. Am. Chem. Soc. *140*, 5987–5999.

10. Li, B., Luo, X., and Dong, Y. (2016). Effects of Chemically Modified Messenger RNA on Protein Expression. Bioconjug. Chem. *27*, 849–853.

11. Kaczmarek, J.C., Kowalski, P.S., and Anderson, D.G. (2017). Advances in the delivery of RNA therapeutics: From concept to clinical reality. Genome Med. *9*, 60.

12. Ramamoorth, M., and Narvekar, A. (2015). Non viral vectors in gene therapy - An overview. J. Clin. Diagn Res. *9*, GE01–GE06.

13. Mo, D., Potter, B.A., Bertrand, C.A., Hildebrand, J.D., Bruns, J.R., and Weisz, O.A. (2010). Nucleofection disrupts tight junction fence function to alter membrane polarity of renal epithelial cells. Am. J. Physiol. Renal Physiol. *299*, F1178-1184.

14. De Queiroz, F.M., Sánchez, A., Agarwal, J.R., Stühmer, W., and Pardo, L.A. (2012). Nucleofection induces non-specific changes in the metabolic activity of transfected cells. Mol. Biol. Rep. *39*, 2187–2194.

15. Van Driessche, A., Ponsaerts, P., Van Bockstaele, D.R., Van Tendeloo, V.F.I., and Berneman, Z.N. (2005). Messenger RNA electroporation: An efficient tool in immunotherapy and stem cell research. Folia Histochem. Cytobiol. *43*, 213–216.

16. Johansson, D.X., Ljungberg, K., Kakoulidou, M., and Liljeström, P. (2012). Intradermal Electroporation of Naked Replicon RNA Elicits Strong Immune Responses. PLoS One *7*, e29732.

17. Bono, N., Ponti, F., Mantovani, D., and Candiani, G. (2020). Non-viral in vitro gene delivery: It is now time to set the bar! Pharmaceutics *12*, 183.

18. Rols, M.-P. (2017). Parameters Affecting Cell Viability Following Electroporation In Vitro. In Handbook of Electroporation, pp. 1449–1465.

19. Kowalski, P.S., Rudra, A., Miao, L., and Anderson, D.G. (2019). Delivering the Messenger: Advances in Technologies for Therapeutic mRNA Delivery. Mol. Ther. *27*, 710–728.

20. Pezzoli, D., Chiesa, R., De Nardo, L., and Candiani, G. (2012). We still have a long way to go to effectively deliver genes! J. Appl. Biomater. Funct. Mater. *10*, 82–91.

21. Xue, H., Guo, P., Wen, W.-C., and Wong, H. (2015). Lipid-Based Nanocarriers for RNA Delivery. Curr. Pharm. Des. *21*, 3140–3147.

22. Moss, K.H., Popova, P., Hadrup, S.R., Astakhova, K., and Taskova, M. (2019). Lipid Nanoparticles for Delivery of Therapeutic RNA Oligonucleotides. Mol. Pharm. *16*, 2265–2277.

23. Jones, C.H., Chen, C.-K., Ravikrishnan, A., Rane, S., and Pfeifer, B.A. (2013). Overcoming Nonviral Gene Delivery Barriers: Perspective and Future. Mol. Pharm. *10*, 4082–4098.

24. Cai, J., Yue, Y., Rui, D., Zhang, Y., Liu, S., and Wu, C. (2011). Effect of chain length on cytotoxicity and endocytosis of cationic polymers. Macromolecules *44*, 2050–2057.

25. Pack, D.W., Hoffman, A.S., Pun, S., and Stayton, P.S. (2005). Design and development of polymers for gene delivery. Nat. Rev. Drug Discov. *4*, 581–593.

26. Rai, R., Alwani, S., and Badea, I. (2019). Polymeric nanoparticles in gene therapy: New avenues of design and optimization for delivery applications. Polymers (Basel). *11*, 745.

27. Nair, J.K., Willoughby, J.L.S., Chan, A., Charisse, K., Alam, M.R., Wang, Q., Hoekstra, M., Kandasamy, P., Kelin, A. V., Milstein, S., et al. (2014). Multivalent N -acetylgalactosamine-conjugated siRNA localizes in hepatocytes and elicits robust RNAi-mediated gene silencing. J. Am. Chem. Soc. *136*, 16958–16961.

28. Nishina, K., Unno, T., Uno, Y., Kubodera, T., Kanouchi, T., Mizusawa, H., and Yokota, T. (2008). Efficient in vivo delivery of siRNA to the liver by conjugation of α-tocopherol. Mol. Ther. *16*, 734–740.

29. Tai, W. (2019). Current aspects of siRNA bioconjugate for in vitro and in vivo delivery. Molecules *24*, 2211.

30. Xia, C.-F., Boado, R.J., and Pardridge, W.M. (2009). Antibody-mediated targeting of siRNA via the human insulin receptor using avidin-biotin technology. Mol. Pharm. *6*, 747–751.

31. Zheng, Y.H., Jeang, K.T., and Tokunaga, K. (2012). Host restriction factors in retroviral infection: Promises in virus-host interaction. Retrovirology *9*, 112.

32. Hatziioannou, T., and Bieniasz, P.D. (2011). Antiretroviral restriction factors. Curr. Opin. Virol. *1*, 526–532.

33. Anderson, J.L., and Hope, T.J. (2005). Intracellular trafficking of retroviral vectors: obstacles and advances. Gene Ther. *12*, 1667–1678.

34. Galla, M., Schambach, A., Towers, G.J., and Baum, C. (2008). Cellular restriction of retrovirus particle-mediated mRNA transfer. J. Virol. *82*, 3069–3077.

35. Prel, A., Caval, V., Gayon, R., Ravassard, P., Duthoit, C., Payen, E., Maouche-Chretien, L., Creneguy, A., Nguyen, T.H., Martin, N., et al. (2015). Highly efficient in vitro and in vivo delivery of functional RNAs using new versatile MS2-chimeric retrovirus-like particles. Mol. Ther. Methods Clin. Dev. *2*, 15039.

36. Kasaraneni, N., Chamoun-Emanuelli, A.M., Wright, G.A., and Chen, Z. (2018). A simple strategy for retargeting lentiviral vectors to desired cell types via a disulfide-bond-forming protein-peptide pair. Sci. Rep. *8*, 10990.

37. Bender, R.R., Muth, A., Schneider, I.C., Friedel, T., Hartmann, J., Plückthun, A., Maisner, A., and Buchholz, C.J. (2016). Receptor-Targeted Nipah Virus Glycoproteins Improve Cell-Type Selective Gene Delivery and Reveal a Preference for Membrane-Proximal Cell Attachment. PLoS Pathog. *12*, e1005641.

38. Buchholz, C.J., Friedel, T., and Büning, H. (2015). Surface-Engineered Viral Vectors for Selective and Cell Type-Specific Gene Delivery. Trends Biotechnol. *33*, 777–790.

39. van der Loo, J.C.M., and Wright, J.F. (2016). Progress and challenges in viral vector manufacturing. Hum. Mol. Genet. *25*, R42–R52.

40. Shirley, J.L., de Jong, Y.P., Terhorst, C., and Herzog, R.W. (2020). Immune Responses to Viral Gene Therapy Vectors. Mol. Ther. *28*, 709–722.
