# Supplementary material for: Improved alpharetrovirus-based Gag.MS2 particles for efficient and transient delivery of CRISPR-Cas9 into target cells
Source: Mol Ther Nucleic Acids. 2022 Jan 1;27:810–23. doi: 10.1016/j.omtn.2021.12.033 (PMC8801357; doi:10.1016/j.omtn.2021.12.033)
Supplement: Document S2. Article plus supplemental information [file mmc2.pdf]

Molecular Therapy
Nucleic Acids
Original Article

Improved alpharetrovirus-based Gag.MS2 particles for efficient and transient delivery of CRISPR-Cas9 into target cells

Yvonne Baron,[1] Johanna Sens,[1] Lucas Lange,[1] Larissa Nassauer,[1] Denise Klatt,[1] Dirk Hoffmann,[1] Marc-Jens Kleppa,[1] Philippe Vollmer Barbosa,[1,2] Maximilian Keisker,[1] Viviane Steinberg,[1] Julia D. Suerth,[1] Florian W.R. Vondran,[3,4] Johann Meyer,[1] Michael Morgan,[1] Axel Schambach,[1,5] and Melanie Galla[1]

[1]Institute of Experimental Hematology, Hannover Medical School, Hannover 30625, Germany; [2]Fraunhofer Institute for Toxicology and Experimental Medicine, Hannover 30625, Germany; [3]ReMediES, Department of General, Visceral and Transplant Surgery, Hannover Medical School, Hannover 30625, Germany; [4]German Centre for Infection Research (DZIF), partner site Hannover-Braunschweig, Hannover Medical School, Hannover 30625, Germany; [5]Division of Hematology/Oncology, Boston Children's Hospital, Harvard Medical School, Boston, MA 02115, USA

DNA-modifying technologies, such as the CRISPR-Cas9 system, are promising tools in the field of gene and cell therapies. However, high and prolonged expression of DNA-modifying enzymes may cause cytotoxic and genotoxic side effects and is therefore unwanted in therapeutic approaches. Consequently, development of new and potent short-term delivery methods is of utmost importance. Recently, we developed non-integrating gammaretrovirus- and MS2 bacteriophage-based Gag.MS2 (g.Gag.MS2) particles for transient transfer of non-retroviral CRISPR-Cas9 RNA into target cells. In the present study, we further improved the technique by transferring the system to the alpharetroviral vector platform (a.Gag.MS2), which significantly increased CRISPR-Cas9 delivery into target cells and allowed efficient targeted knockout of endogenous *TP53/Trp53* genes in primary murine fibroblasts as well as primary human fibroblasts, hepatocytes, and cord-blood-derived CD34^{+} stem and progenitor cells. Strikingly, co-packaging of *Cas9* mRNA and multiple single guide RNAs (sgRNAs) into a.Gag.MS2 chimera displayed efficient targeted knockout of up to three genes. Co-transfection of single-stranded DNA donor oligonucleotides during CRISPR-Cas9 particle production generated all-in-one particles, which mediated up to 12.5% of homology-directed repair in primary cell cultures. In summary, optimized a.Gag.MS2 particles represent a versatile tool for short-term delivery of DNA-modifying enzymes into a variety of target cells, including primary murine and human cells.

INTRODUCTION

Gene and cell therapies are promising for the treatment of inherited or acquired diseases and are already successfully used clinically to combat hematological cancers and to treat monogenic diseases.[1–3] Here, the therapeutic gene is provided in an additive fashion that is stably integrated into the genome, e.g., by retrovirus-derived vectors or transposons.[4,5] However, despite the clear clinical benefit, some retrovirus-based trials were overshadowed by adverse events in the form of leukemia caused by unexpected proto-oncogene activation by insertional mutagenesis.[6–9] Also, retroviral gene delivery with artificial expression cassettes often does not mediate physiologic gene expression. Precise gene-editing technologies can be applied to correct disease-causing mutations and/or removal of deleterious genome sequences and thus circumvent both insertional mutagenesis and dysregulated gene expression.[10]

Currently, the most frequently used gene-editing platforms are transcription activator-like effector (TALEN), zinc finger (ZFN), and RNA-guided CRISPR-Cas nucleases, among which CRISPR-Cas9 has become the most popular and convenient gene-editing technology.[11,12] Here, CRISPR stands for clustered regularly interspaced short palindromic repeats, which are DNA sequences that, together with CRISPR-associated (Cas) nuclease genes, originate from the adaptive immune system of bacteria and archaea.[13,14] Further work has fine-tuned the technology for application to gene-editing approaches in eukaryotic cells, including human, with the result that CRISPR-Cas9 has become an indispensable tool in biological research and clinical trials, for example, for hemoglobinopathies.[15]

However, like other DNA-modifying enzymes, CRISPR-Cas9 also has limitations and should be expressed in a dose-controlled manner, as constitutive expression of CRISPR-Cas9 components was shown to result in off-target events and to inhibit cell proliferation, even in the absence of a co-expressed single guide RNA (sgRNA).[16] Moreover,

Received 7 July 2020; accepted 29 December 2021;
https://doi.org/10.1016/j.omtn.2021.12.033.

Correspondence: Melanie Galla, PhD, Institute of Experimental Hematology, Hannover Medical School, Carl-Neuberg-Strasse 1, Hannover 30625, Germany.
E-mail: galla.melanie@mh-hannover.de
Correspondence: Axel Schambach, MD, PhD, Institute of Experimental Hematology, Hannover Medical School, Carl-Neuberg-Strasse 1, Hannover 30625, Germany.
E-mail: schambach.axel@mh-hannover.de

This is an open access article under the CC BY license (http://creativecommons.org/licenses/by/4.0/).

even temporary expression of CRISPR-Cas9 components or ZFN from plasmid DNA or integrase-deficient lentiviral vectors showed off-target activity and resulted in integrated DNA fragments at on-target and off-target sites in the genome.[17–21] Therefore, DNA-modifying enzymes should preferably be delivered as a "hit-and-run" strategy, for example, as RNAs and/or proteins to increase the safety of the gene-editing procedure. Indeed, electroporated Cas9 protein and/or *in vitro* transcribed *Cas9* mRNA together with the sgRNA of interest minimized these unwanted side effects.[17,22] However, such conventional delivery methods require delivery of RNA or protein in enormous excess and are still associated with voltage-induced cytotoxicity.[23,24] Furthermore, other innovative gene-delivery strategies, such as lipid- or polymer-based non-viral vectors, may also be associated with a substantial degree of cytotoxicity and/or suffer from inefficient endosomal escape of the delivered nucleic acid cargo.[25–28]

Therefore, the development of novel and improved RNA and/or protein delivery methodologies is of great interest. One possibility is the use of non-integrating reverse transcription disabled retroviral vectors,[29–34] as they—like their integrating wild-type (wt) counterparts—not only possess all retroviral properties for efficient and non-toxic cell entry but can also be specifically retargeted to any desired cell type by innovative pseudotyping strategies.[35–38]

Recently, we and others developed retrovirus-based MS2 bacteriophage (MS2) chimera for reversible delivery of the multi-component CRISPR-Cas9 technology into target cells.[16,39–41] Redirecting the retroviral packaging machinery to respective components of MS2 allowed us to specifically deliver RNA species other than mRNA, including RNA polymerase III (RNA Pol III)-driven transcripts.[16] Similar to retroviruses, MS2 is a plus- and single-stranded RNA virus, whose genome is embedded within a capsid formed by the viral MS2 coat protein (MS2CP). During particle formation, MS2CP acts as a dimer and binds the MS2 RNA genome via a small target site (TS), which is a 23-nt-long hairpin structure, thereby ensuring its encapsidation.[42] We explored the MS2 packaging mechanism in the context of a gammaretroviral vector system and generated murine leukemia virus (MLV)-based/MS2 virus-like particles, in which the nucleocapsid (NC) domain of the gammaretroviral structural group-specific antigen (Gag) polyprotein was substituted with a genetically fused MS2CP dimer[43] (g.Gag.MS2) to deliver non-retroviral *SpCas9* mRNA and sgRNA transcripts.[44,45] Co-packaging of *SpCas9* mRNA and sgRNA resulted in the generation of potent and significantly less cytotoxic g.Gag.MS2.CRISPR-Cas9 all-in-one (g.Gag.MS2.CRISPR) particles, which induced efficient knockout of several genes in various cell types.[16] Moreover, the spatiotemporal co-delivery of CRISPR-Cas9 components by g.Gag.MS2.CRISPR particles strongly reduced the amount of RNA necessary to achieve high knockout rates as compared with other RNA transfer methods.

In the present study, we aimed to further improve the potency of this approach and transferred the gammaretroviral MLV-based technology to the recently described alpharetroviral Rous sarcoma virus (RSV)-based vector platform.[46] Side-by-side comparison of newly generated alpharetroviral Gag.MS2 (a.Gag.MS2) particles with g.Gag.MS2 particles revealed enhanced CRISPR-Cas9 delivery into target cells. These particles allowed efficient targeted knockout of endogenous *TP53/Trp53* genes in several primary cell sources. Noteworthy, co-packaging of *SpCas9* mRNA and multiple sgRNA transcripts into a.Gag.MS2 chimera showed efficient, simultaneous, and targeted knockout of up to three genes. Co-transfection of a single-stranded DNA oligonucleotide (ssODN) during CRISPR-Cas9 particle production led to all-in-one particles, capable of homology-directed repair (HDR) in HT1080 and primary cell cultures. In summary, we report optimized a.Gag.MS2 particles as a versatile tool for short-term and dose-controlled delivery of CRISPR-Cas9 into target cells.

RESULTS

The concept of g.Gag.MS2.CRISPR particles

The recently described g.Gag.MS2.CRISPR particles were designed for spatiotemporal co-delivery of *Cas9* and sgRNA transcripts into target cells. The preservation of most Gag subdomains should allow the generation of retrovirus-like particles, which protect the non-retroviral RNA cargo from extracellular nucleases and facilitate, together with the envelope glycoprotein, introduction of the RNA cargo into cells in a non-toxic manner.

To realize this concept, we designed a g.Gag.MS2 precursor protein variant, in which the NC was substituted by a genetically fused MS2CP dimer that was separated from the Gag capsid (CA) subdomain by the natural retroviral protease site (Figure 1A). In addition, we generated non-retroviral expression plasmids encoding TS-containing *Streptococcus pyogenes* Cas9 (*SpCas9.TS*) mRNA and RNA-Pol-III-driven sgRNA.TS transcripts, which contain the TS hairpins incorporated either within (TS.inc sgRNA) or adjacent (TS.adj sgRNA) to the sgRNA scaffold.[47,48] For g.Gag.MS2.CRISPR particle production, producer cells were co-transfected with g.Gag.MS2, SpCas9.TS, and TS.adj or TS.inc sgRNA encoding plasmids and an expression plasmid for the envelope glycoprotein from vesicular stomatitis virus G protein (VSV-G). Direct comparison of TS.inc or TS.adj sgRNA-containing g.Gag.MS2.CRISPR particles targeting the mouse *Tet methylcytosine dioxygenase* 2 (*Tet2*) gene resulted in efficient knockout rates of a far-red fluorescent *RFP657* reporter gene that contained the *Tet2* recognition site downstream of its start codon (*RFP657.Tet2*; Figures 1B and S1A). Strikingly, knockout rates by both g.Gag.MS2.CRISPR.Tet2 particle variants were comparable to those mediated by a stable integrating retroviral transfer vector (g.RIT.CRISPR.Tet2; Figure S1B) constitutively expressing (no TS-carrying) *SpCas9* and Tet2 sgRNA transcripts. Interestingly, g.Gag.MS2.CRISPR.Tet2 particles containing the Tet2.TS.adj sgRNA variant were slightly more (~1.5-fold) efficient than respective Tet2.TS.inc sgRNA particles when equal supernatant volumes were compared.

Generation and validation of a.Gag.MS2.CRISPR particles

The recently designed g.Gag.MS2.CRISPR particles showed efficient and reversible delivery of CRISPR-Cas9 components into various target cells.[16] However, relatively high volumes of 100-fold

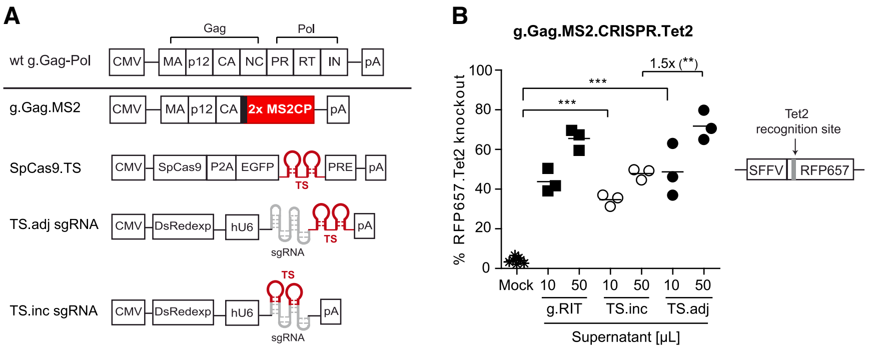

Figure 1. Gammaretrovirus-based Gag.MS2 chimera for CRISPR-Cas9 delivery

(A) Design of the gammaretroviral Gag.MS2 (g.Gag.MS2) variant and non-retroviral *SpCas9.TS* and sgRNA.TS expression plasmids. The configuration of structural Gag and enzymatic Pol proteins within gammaretroviral wt Gag-Pol (wt g.Gag-Pol) is depicted at the top. It consists of matrix (MA), p12, capsid (CA), nucleocapsid (NC), protease (PR), reverse transcriptase (RT), and integrase (IN) subdomains, which are separated from each other by individual protease sites. In g.Gag.MS2, the MS2CP dimer (2× MS2CP) protein replaces NC while maintaining the natural retroviral protease site (black bold bar). To ensure specific packaging of CRISPR-Cas9 RNA into assembling g.Gag.MS2 particles, two copies of MS2 target site (TS) are incorporated downstream of *EGFP* within the *SpCas9.TS* expression plasmid or were placed in (TS.inc) or adjacent (TS.adj) to the sgRNA scaffold in respective sgRNA expression plasmids. Co-expression of *EGFP* and *DsRedexp* helped to monitor transfection during particle production. (B) Direct comparison of integrating (g.RIT) and non-integrating g.Gag.MS2 CRISPR.Tet2 particles. Indicated supernatant volumes were used to transduce human HT1080-based RFP657.Tet2 reporter cells. Each data point represents one individually generated supernatant (n = 3). CMV, promoter from cytomegalovirus; hU6, human RNA Pol III U6 promoter; pA, poly(A) signal; PRE, post-transcriptional regulatory element from woodchuck hepatitis virus; SFFV, promoter from spleen focus forming virus.

concentrated supernatant (50 μL) were needed to achieve robust knockout rates ≥60% with particles containing the slightly better performing Tet2.TS.adj sgRNA variant (Figure 1B). Therefore, we aimed to further improve the technique, as an enhanced potency would save resources and enable a broader range of applications, especially with respect to the manipulation of primary cells.

As several retroviruses have co-evolved in different vertebrate hosts over millions of years,[49–51] they have acquired species-specific mutations and susceptibilities to cellular antiviral restriction factors and adapted to use different cellular co-factors.[52–54] Thus, we hypothesized that switching the gammaretrovirus-based vector platform to the phylogenetically remote alpharetroviral genus might be beneficial for Gag.MS2 particle generation and could overcome potential target-cell-related restrictions.

We generated an avian RSV-based a.Gag.MS2 variant, in which the MS2CP dimer replaces the protease, reverse transcriptase, and integrase subdomains of wt a.Gag-Pol and was separated from NC with sequences of the natural retroviral protease site (Figure 2A). Since the co-packaging of TS.adj sgRNA (compared with TS.inc) was found to increase the activity of g.Gag.MS2.CRISPR particles (Figure 1B),[16] we initially elucidated which sgRNA.TS variant is best suited for a.Gag.MS2.CRISPR particle production (Figure 2B). We generated *Tet2*-targeting a.Gag.MS2.CRISPR (a.Gag.MS2.CRISPR.Tet2) particles carrying either Tet2.TS.inc or Tet2.TS.adj sgRNAs and transduced human HT1080-based RFP657.Tet2 reporter cells.[16,55] Surprisingly, and in contrast to g.Gag.MS2.CRISPR particles, a.Gag.MS2.CRISPR particles that were generated in the presence of the Tet2.TS.inc sgRNA showed up to 5.4-fold enhanced *RFP657.Tet2* knockout rates compared with Tet2.TS.adj sgRNA-containing particles. Remarkably, the application of only 0.5 μL of a.Gag.MS2.CRISPR.Tet2.TS.inc supernatant yielded mean *RFP657.Tet2* knockout rates of 46%. As this was 20-fold less supernatant volume than what was needed for comparable knockout rates mediated by Tet2.TS.adj-containing g.Gag.MS2.CRISPR particles (compare Figures 2B and 1B), we also directly compared the best-working Gag.MS2.CRISPR.Tet2 particle configuration of both retroviral genera. To simplify the nomenclature of a.Gag.MS2.CRISPR.Tet2.TS.inc and g.Gag.MS2.CRISPR.Tet2.TS.adj and all other CRISPR-Cas9 particles, the position of TS within the sgRNA backbone will not be specifically displayed in the following sections.

Application of identical volumes of a.Gag.MS2.CRISPR.Tet2 or g.Gag.MS2.CRISPR.Tet2 supernatants to HT1080 RFP657.Tet2 reporter cells showed that a.Gag.MS2-based particles significantly outperformed their g.Gag.MS2-based correlate with ∼4- to 26-fold higher *RFP657.Tet2* knockout rates (Figure 2C). In these experiments, 5 μL of a.Gag.MS2.CRISPR.Tet2 supernatant was sufficient to achieve robust average knockout rates >70%.

The applicability of both particle types was next tested in human induced pluripotent stem cells (hiPSCs), which represent a promising cell source for basic and clinical research.[56] Therefore, we engineered an hiPSC line to express the *RFP657.Tet2* reporter gene from a silencing-resistant promoter consisting of the minimal ubiquitous chromatin opening element CBX3 fused with the elongation factor 1 alpha short (EFS) enhancer/promoter sequences (Figure S1A).[57,58] In accordance with the results in HT1080-based RFP657.Tet2 reporter cells, a.Gag.MS2.CRISPR.Tet2 supernatants were more effective than g.Gag.MS2.CRISPR.Tet2 supernatants and yielded *RFP657.Tet2* knockout rates in hiPSC of >50% with 5 μL applied supernatant (Figure 2D). Interestingly, lentiviral integrating transfer (LIT) CRISPR.Tet2 (Figure S1B) control particles that usually perform well in hiPSC[57] showed only moderate knockout rates of 26% (2.9 μL = MOI 2) or 35% (7.4 μL = MOI 5). Since CRISPR-Cas9 treatment was shown to be highly cytotoxic in hiPSCs,[59] we additionally determined the total number of cells 5 days post-transduction (Figure S2A). In comparison to untreated mock cultures, we found reduced cell numbers in all treated cultures independent of the CRISPR-Cas9

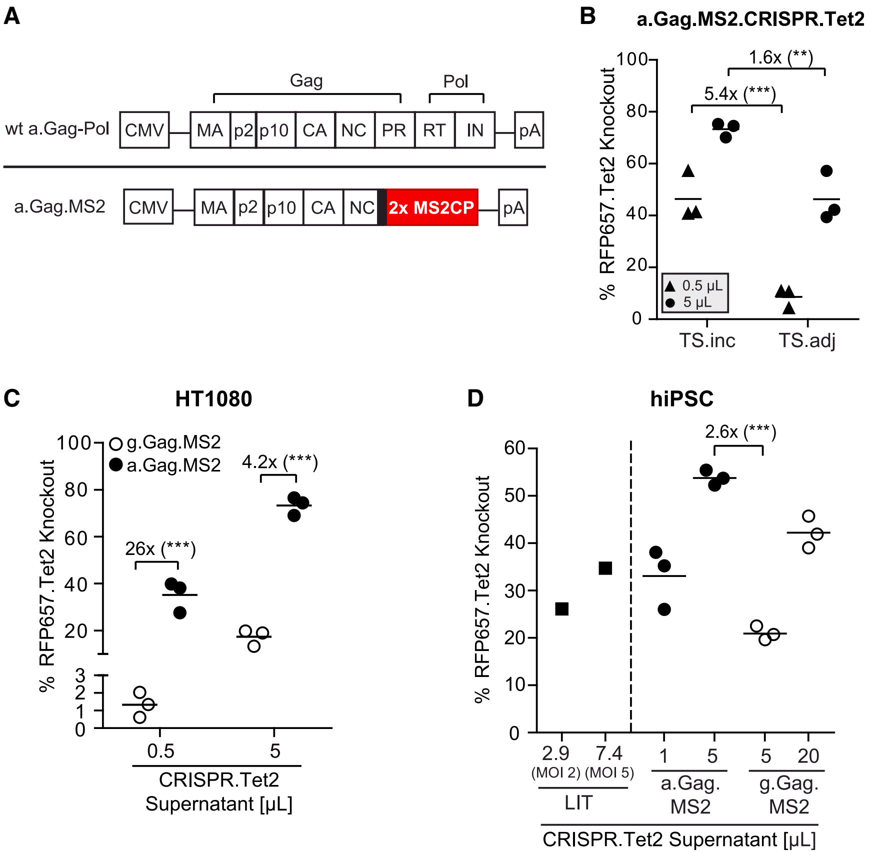

Figure 2. Alpharetrovirus-based Gag.MS2 particles showed improved delivery of CRISPR-Cas9 components into target cells

(A) Design of alpharetroviral Gag.MS2 (a.Gag.MS2) variants. The wt alpharetroviral Gag-Pol polyprotein (wt a.Gag-Pol) is shown at the top of the figure panel. In contrast to g.Gag-Pol, alpharetroviral PR is part of the Gag open reading frame (ORF) (and not Pol ORF). In the depicted a.Gag.MS2 variant, the MS2CP dimer was separated from NC by the naturally occuring viral protease site (black bold bar). (B) a.Gag.MS2.CRISPR.Tet2 particles containing the Tet2.TS.inc sgRNA variant depict higher knockout rates compared with their Tet2.TS.adj counterparts. Indicated volumes of supernatants were used to transduce HT1080-based RFP657.Tet2 reporter cells. The graph depicts *RFP657.Tet2* knockout rates mediated by three independent supernatants (n = 3). (C) a.Gag.MS2.CRISPR.Tet2 particles are superior to their gammaretroviral counterparts. Direct comparison of three individually produced a.Gag.MS2- and g.Gag.MS2-based CRISPR.Tet2 supernatants in HT1080-based RFP657.Tet2 reporter cells is shown (n = 3). (D) a.Gag.MS2.CRISPR.Tet2 particles mediate efficient targeted gene knockout in hiPSCs. hiPSC-based RFP657.Tet2 reporter cells were transduced with indicated volumes of three different batches of g.Gag.MS2.CRISPR.Tet2 or a.Gag.MS2.CRISPR.Tet2 supernatants (n = 3). A LIT.CRISPR.Tet2 supernatant applied at MOI 2 (2.9 µL) or MOI 5 (7.4 µL) served as a positive control (n = 1).

delivery method. However, the loss of viable cells was less pronounced in Gag.MS2-treated cultures and increased with the amount of the applied volume, suggesting reduced cytotoxicity by low and time-limited expression of CRISPR-Cas9 components in hiPSC. Noteworthy, the expression of the pluripotency marker TRA-1-60 was not altered in any of the tested conditions (Figure S2B).

In summary, the transfer of the Gag.MS2-based technology from the gamma- to the alpharetroviral background significantly improved its efficiency and resulted in enhanced CRISPR-Cas9-mediated knockout rates in human fibroblasts as well as hiPSC. Furthermore, the non-integrating nature of Gag.MS2 particles seemed to be beneficial in hiPSC, as they outperformed their integrating LIT correlates in terms of (knockout) efficiency and cytotoxicity ratios.

Characterization of g.Gag.MS2 and a.Gag.MS2 CRISPR.Tet2 supernatants

The comparative functional analyses of alpha- and gammaretroviral Gag.MS2.CRISPR.Tet2 supernatants revealed a superior performance for a.Gag.MS2-based preparations (Figures 2C and 2D). To better understand the underlying mechanisms, we produced in total six supernatants (two different time points, three supernatants each) for both a.Gag.MS2 and g.Gag.MS2 CRISPR.Tet2 particles and characterized them in more detail with regard to the content of CRISPR-Cas9 components and Gag.MS2 precursor proteins (Figure 3). Overall, we determined more Tet2.TS sgRNA than *SpCas9.TS* mRNA transcripts in respective supernatants (Figure 3A). However, a.Gag.MS2.CRISPR.Tet2 supernatants had 1.7×10^7 copies/µL supernatant, which corresponds to 8-fold-higher *SpCas9.TS* mRNA levels than their gammaretroviral correlate (2×10^6 copies/µL). Conversely, g.Gag.MS2-based supernatants had 2.3-fold-more Tet2.TS sgRNA than a.Gag.MS2.CRISPR.Tet2 supernatants. Interestingly, in both types of supernatants, we detected SpCas9 protein, whose levels varied between supernatant batches (Figure 3B). Next, we performed immunoblot analysis of all three CRISPR.Tet2 supernatants from the same batch (batch 2; see black filled circles in Figures 3A and 3B) and semi-quantitatively determined the Gag.MS2 precursor protein levels in these supernatants (Figure 3C). In line with higher *SpCas9.TS* mRNA concentrations, we also found more Gag.MS2 protein in a.Gag.MS2 supernatants compared with their gammaretroviral counterparts. However, compared with the 8-fold difference in *SpCas9.TS* mRNA content, the difference in Gag.MS2 protein precursor levels was only 4-fold.

Having determined the content of CRISPR components and Gag.MS2 precursor proteins within a.Gag.MS2 and g.Gag.MS2 CRISPR.Tet2 supernatants, we wanted to more thoroughly assess their potency to knockout *RFP657.Tet2* in HT1080-based reporter cells. We used

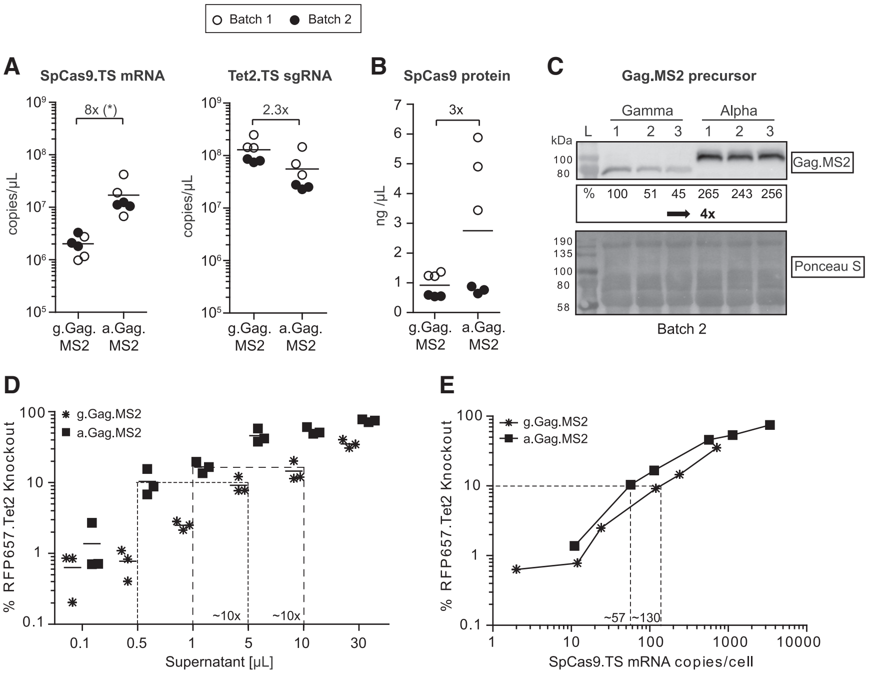

Figure 3. Characterization of alpha- and gammaretroviral Gag.MS2.CRISPR.Tet2 supernatants

In total, two different batches (white and black filled circles), each with three individually packaged supernatants for a.Gag.MS2 and g.Gag.MS2 CRISPR.Tet2 particles, were generated and characterized for their content of CRISPR-Cas9 components (n = 6). (A) *SpCas9.TS* mRNA and sgRNA.TS content in a.Gag.MS2- and g.Gag.MS2-based CRISPR.Tet2 supernatants. Total RNA copies per microliter supernatant were calculated using individual plasmid standards. (B) Gag.MS2.CRISPR.Tet2 supernatants contain SpCas9 protein. SpCas9 protein concentrations were assessed by an SpCas9 ELISA. (C) a.Gag.MS2.CRISPR.Tet2 supernatants had a higher Gag.MS2 precursor protein content. Immunoblot analysis of three individually packaged a.Gag.MS2.CRISPR.Tet2 and g.Gag.MS2.CRISPR.Tet2 supernatants is shown (batch 2; black filled circles) (n = 3). g.Gag.MS2 (82 kDa) and a.Gag.MS2 (89 kDa) precursor proteins were detected with an anti-MS2 antibody. Ponceau S staining of the membrane is shown below. (D) g.Gag.MS2.CRISPR.Tet2-mediated *RFP657.Tet2* knockout rates can be adjusted by ~10-fold higher supernatant volumes. Characterized supernatants from batch 2 were used to transduce HT1080 RFP657.Tet2 reporter cells at the depicted volumes (n = 3). (E) Normalization of supernatant volumes showed an ~2-fold-higher potency for a.Gag.MS2-based CRISPR.Tet2 particles. The in (D) acquired datasets were replotted as % *RFP657.Tet2* knockout against applied *SpCas9.TS* mRNA copies/cell (n = 3; mean values are depicted).

the same supernatant batches that were also subjected to immunoblot analysis and determined the *RFP657.Tet2* knockout rates for the indicated increasing amounts of supernatant volumes (Figure 3D). In line with the 8-fold-higher *SpCas9.TS* mRNA content in a.Gag.MS2.CRISPR.Tet2 supernatants, ~10-fold-more g.Gag.MS2 supernatant volume was needed to achieve similar *RFP657.Tet2* knockout rates. Graphical analysis of *RFP657.Tet2* knockout rates against applied *SpCas9.TS* mRNA copies per cell (Figure 3E) showed similar genome-editing potency per delivered *SpCas9.TS* mRNA. However, we still observed a maximally ~2.3-fold difference between both particle types, with a.Gag.MS2 being more potent than g.Gag.MS2 particles when the same amount of *SpCas9.TS* mRNA was applied. For example, transduction of HT1080 RFP657.Tet2 reporter cells with a.Gag.MS2 supernatant volumes corresponding to ~57 *SpCas9.TS* mRNA copies per cell resulted in *RFP657.Tet2* knockout rates of ~10%. In comparison, ~130 copies/cell of *SpCas9.TS* mRNA had to be transferred by g.Gag.MS2 particles to give the same *RFP657.Tet2* knockout levels.

Taken together, a.Gag.MS2.CRISPR.Tet2 supernatants contain more *SpCas9.TS* vector mRNA and Gag.MS2 precursor proteins, which indicate higher functional titers and explain their better performance. Therefore, the upcoming experiments were exclusively performed with a.Gag.MS2-based CRISPR-Cas9 particles. As the content of *SpCas9.TS* mRNA is the best parameter that reflects the potency of respective Gag.MS2 supernatants, a.Gag.MS2 supernatants were titrated based on this CRISPR-Cas9 component prior to usage.

Simultaneous knockout of multiple genes by a.Gag.MS2.CRISPR particles

Simultaneous knockout of more than one gene is desired for some applications. Since supernatants with a.Gag.MS2.CRISPR.Tet2 particles contain high amounts of Tet2.TS sgRNA transcripts (~6 $\times$ 10^7 copies/µL; Figure 3A), we next asked whether it is possible to co-package multiple gene-targeting sgRNA.TS variants into the same particle. To test this hypothesis, we initially generated five individual a.Gag.MS2.CRISPR all-in-one supernatants in the presence of two different sgRNA.TS expression plasmids, encoding protospacer sequences targeting either *CXCR4* or *Tet2* genes. To more closely evaluate the distribution of CRISPR RNA within these particles, in addition to the abundance of *SpCas9.TS* mRNA, we also determined the content of CXCR4.TS and Tet2.TS sgRNA transcripts in these supernatants (Figure 4A). As seen for a.Gag.MS2.CRISPR.Tet2 particle supernatants with just one sgRNA (Figure 3A), dual gene-targeting supernatants also revealed more sgRNA.TS than *SpCas9.TS* transcripts. Interestingly, co-expression of an additional sgRNA.TS variant during packaging reduced *SpCas9.TS* mRNA levels in supernatants ~7-fold (compare Figures 4A and 3A). To test the efficacy of these supernatants, we used a human Jurkat T cell clone that was sorted for high endogenous *CXCR4* expression[16] and additionally modified to co-express the *RFP657.Tet2* reporter gene (Jurkat CXCR4/RFP657.Tet2 reporter cells; Figure 4B). We applied 250, 500, or 1,250 *SpCas9.TS* mRNA copies/cell and achieved efficient simultaneous *Tet2* and *CXCR4* knockout of ~37%–53%. Next, we produced multiplexing a.Gag.MS2.CRISPR particles containing CXCR4.TS, Tet2.TS, and EGFP.TS sgRNA transcripts and transduced Jurkat cells

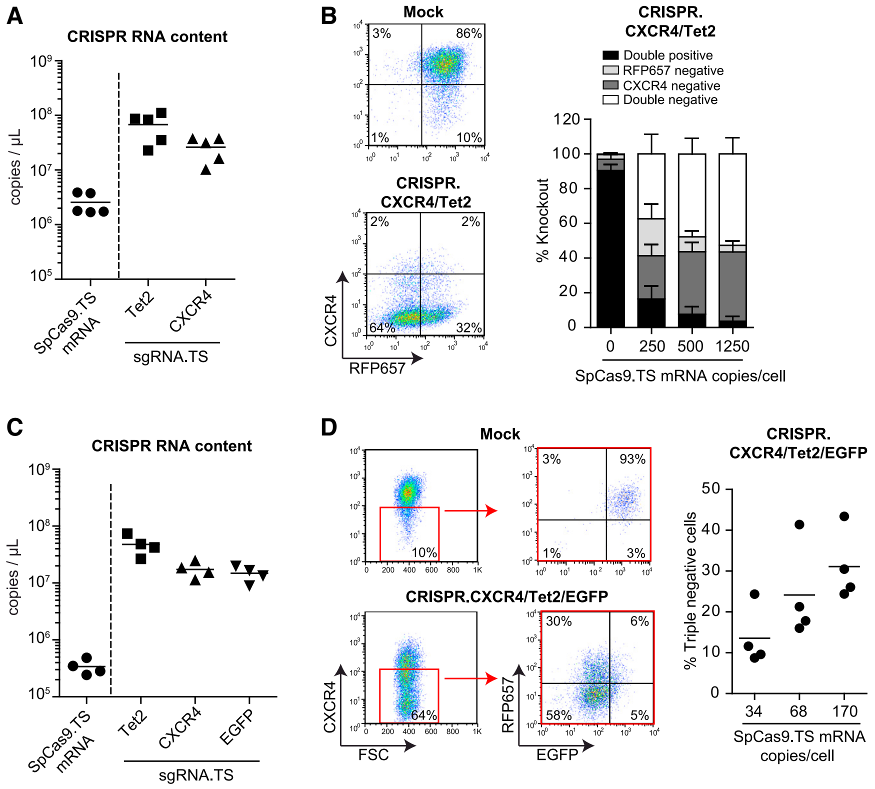

Figure 4. Multiplexing with a.Gag.MS2.CRISPR particles

(A–D) Efficient simultaneous knockout of two (B) or three (D) target genes by a.Gag.MS2.CRISPR particles. Prior to transduction of CXCR4^{+} Jurkat cells co-expressing *RFP657.Tet2* and/or *EGFP*, the CRISPR RNA content of supernatants containing a.Gag.MS2 particles with two (A) or three (C) sgRNA.TS variants was determined. In total, knockout data of 4 to 5 individually packaged supernatants are depicted (n = 4–5). Representative flow cytometry plots of untransduced cells (mock) and of cells that were treated with the highest applied particle dose of the respective a.Gag.MS2.CRISPR supernatants are also shown. Errors bars in (B) indicate standard deviations.

that were triple positive for these target genes. Prior to particle application, we also determined *SpCas9.TS* mRNA and sgRNA.TS transcript concentrations in these supernatants and found similar sgRNA.TS, but again ~7- to 50-fold-lower *SpCas9.TS* mRNA transcript levels compared with dual or mono-targeting supernatants, respectively (compare Figures 3A, 4A, and 4C). Remarkably, the treatment of cells with the indicated particle doses resulted in average triple knockout efficiencies of ~14% (34 copies/cell), ~24% (68 copies/cell), and ~31% (136 copies/cell; Figure 4D).

Knockout of endogenous *TP53/Trp53* genes in primary cells by a.Gag.MS2.CRISPR particles

We next tested the applicability of our a.Gag.MS2.CRISPR particles in human and murine primary cells. We chose to knockout human or mouse endogenous tumor suppressor genes *TP53* (human) or *Trp53* (murine), respectively, as altered expression of the p53 master regulator protein may promote cell survival and division, which would facilitate our experimental readout.[60,61] Therefore, we generated several batches of a.Gag.MS2.CRISPR supernatants comprising particles carrying either TP53.TS or Trp53.TS sgRNA variants and quantified their CRISPR RNA content (Figure S3). As for all analyzed Gag.MS2.CRISPR supernatants (Figures 3 and 4) so far, a.Gag.MS2-based CRISPR.TP53 and CRISPR.Trp53 preparations revealed more sgRNA.TS than *SpCas9.TS* transcripts. Regarding absolute *SpCas9.TS* mRNA numbers, both supernatant types showed on average 8.3×10^{6} (TP53) or 1.2×10^{7} (Trp53) *SpCas9.TS* mRNA copies/µL.

First, we transduced human newborn foreskin fibroblasts (NuFFs) with indicated increasing doses of a.Gag.MS2.CRISPR.TP53 supernatants and assessed the *TP53* insertion and deletion (indel) frequencies within these cultures via tracking of indels by decomposition (TIDE) analysis.[62] Strikingly, the lowest applied particle dose with ~83 *SpCas9.TS* mRNA copies/cell already yielded in *TP53* knockout rates of ~13%, which could be further increased to ~93% knockout with the highest particle dose used (Figure 5A). To test whether the measured *TP53* knockout had a biological effect, we monitored cell proliferation of treated cultures over time (Figure 5B). Compared with non-treated mock cultures, we observed an increase in cell numbers in cultures treated with a.Gag.MS2.CRISPR.TP53 supernatants, which strongly correlated with determined *TP53* indel rates. Next, we tested a.Gag.MS2.CRISPR.TP53 supernatants on primary human hepatocytes (PHHs) and primary human umbilical-cord-blood-derived CD34^{+} hematopoietic stem and progenitor cells (hCD34^{+} HSPCs). Based on the results in NuFF cells, we applied particle doses up to 4,150 *SpCas9.TS* mRNA copies/cell. Remarkably, determined *TP53* knockout rates in treated PHH cultures were as efficient as those that we measured in transduced NuFF cultures (Figure 5C). In contrast, *TP53* knockout rates in hCD34^{+} HSPCs were moderate as we achieved indel rates ranging from 6% to 36% (Figure 5D). Finally, we investigated the efficacy of the technique in CF1 and C3H murine embryonic fibroblasts (MEFs) (Figure 5E). As preliminary experiments showed that alpharetroviral wt RIT vector particles are slightly more restricted in murine iPSCs than their gammaretroviral counterparts, we applied 4,800 *SpCas9.TS* mRNA copies/cell on 2 consecutive days and obtained mean *Trp53* indel rates of 31% (CF1) and 25% (C3H).

a.Gag.MS2.CRISPR particles mediate HDR in human HT1080 and NuFF fibroblasts

Finally, we investigated whether the significantly improved a.Gag.MS2.CRISPR particles are also suitable for CRISPR-Cas9-mediated HDR. To test this, we used a previously described fluorescence transition model, in which *EGFP* is converted to enhanced blue fluorescent

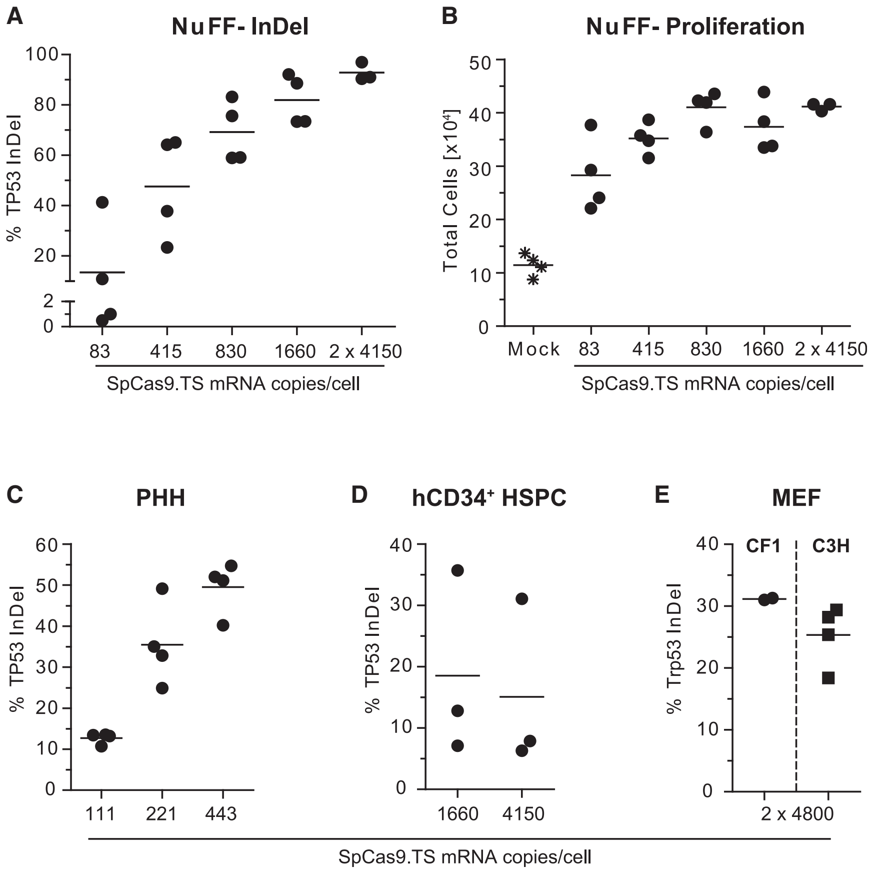

Figure 5. Efficient gene editing of primary cells by a.Gag.MS2.CRISPR particles

(A) Highly efficient knockout of endogenous *TP53* in primary human NuFF cells. NuFF cells were transduced with serial dilutions of a.Gag.MS2.CRISPR.TP53 particles reflecting the indicated particle doses expressed as *SpCas9.TS* mRNA copies per cell. The knockout efficiencies were determined by TIDE analysis. (B) *TP53* knockout cells showed enhanced proliferation compared with non-treated mock cells. Cells from (A) were counted and seeded at a density of 1×10^4 per well. Seven to eight days later, the respective total number of cells was determined. (C and D) *TP53* knockout rates in primary human hepatocytes (PHHs) and human umbilical cord blood-derived $CD34^+$ hematopoietic stem and progenitor cells (h$CD34^+$ HSPCs). (E) Efficient knockout of endogenous *Trp53* in murine embryonic fibroblasts (MEFs). CF1 or C3H MEFs were transduced with 4,800 *SpCas9.TS* mRNA copies/cell on 2 consecutive days. The data points in respective graphs reflect biological replicates with independently prepared supernatants (n = 2–4).

protein (*EBFP*) by the substitution of two amino acids (T66S and Y67H).[63] Respective a.Gag.MS2.CRISPR.HDR particles were produced in the presence of sgRNA.TS transcripts, designed to target the relevant site in *EGFP*, and no TS-carrying ssODN donor templates encoding the T66S and Y67H mutations, including flanking homology arms and with or without a stabilizing phosphorothioate (−PT or +PT) bond modification (Figure S4A).[64,65] To avoid cutting by the SpCas9 enzyme, the protospacer adjacent motif (PAM) within ssODNs was destroyed by the introduction of a silent point mutation. In addition, we engineered human HT1080 cells to express *EGFP* via lentiviral transduction. Quantitative real-time PCR analysis of genomic DNA from this bulk culture revealed a mean vector copy number (mVCN) of ~1. Independent of the co-packaged −PT or +PT ssODN, transduction of EGFP-expressing HT1080 cells with a.Gag.MS2.CRISPR.HDR particles resulted in comparable and efficient *EGFP* knockout rates of up to ~56% (Figure S4B). Determination of the percentage of $EBFP^+$ cells within the $EGFP^-$ population revealed successful HDR for −PT and +PT ssODN a.Gag.MS2.CRISPR.HDR particles, especially with high supernatant volumes (50–100 μL). However, the use of PT-stabilized ssODNs significantly increased HDR events and resulted in HDR rates of up to 5% already with 30 μL applied supernatant. To further confirm these results, we analyzed the performance of a.Gag.MS2.CRISPR.HDR supernatants in the presence of non-homologous end joining (inh.NHEJ; M3814)[66] or HDR (inh.HDR; YU238259)[67] DNA repair inhibitors (Figure 6). For this purpose, we prepared four new a.Gag.MS2.CRISPR.HDR supernatants and determined their *SpCas9.TS* mRNA, EGFP.TS sgRNA, and ssODN content (Figure 6A). Interestingly, supernatants contained similar levels of *SpCas9.TS* mRNA (6.6×10^6 copies/μL) and ssODN template (3.6×10^6 copies/μL), while the EGFP.TS sgRNA transcript levels were on average 6.1-fold higher (3.1×10^7 copies/μL). Subsequently, we transduced HT1080 EGFP reporter cells with 30 μL (~1,980 *SpCas9.TS* mRNA copies/cell) a.Gag.MS2.CRISPR.HDR supernatant in the absence (w/o) or presence of DMSO, inh.NHEJ, or inh.HDR (Figure 6B). In line with the previous experiments (Figure S4A), freshly produced a.Gag.MS2.CRISPR.HDR supernatants showed similar percentages of $EBFP^+$ cells in treated cultures, which could be further increased or suppressed by the application of the NHEJ or HDR inhibitors, respectively. To show successful HDR on the genetic level, $EBFP^+$ / $EGFP^-$ cells were sorted from three cultures that were treated with a.Gag.MS2.CRISPR.HDR particles (Figure S5A). Analyses of the sequencing results covering the relevant transgene region with the help of the ICE v.2 CRISPR analysis tool from Synthego[68] revealed *EBFP* sequences and ssODN knockin rates of 54%–61% (Figure S5B). Surprisingly, sequencing results showing clear conversion of *EGFP* to *EBFP* revealed ambiguous sequencing results comprising the alteration of PAM. Finally, we tested the same dose of a.Gag.MS2.CRISPR.HDR particles on EGFP-expressing primary human NuFF cells (Figure 6C). In these cells, which possess a mVCN of ~5, we achieved up to 12.5% (mean ~11%) $EBFP^+$ cells. Blocking NHEJ did not further increase HDR, but inhibition of HDR reduced the occurrence of $EBFP^+$ cells to almost background levels. Subsequent molecular analyses of selected samples confirmed the transformation of

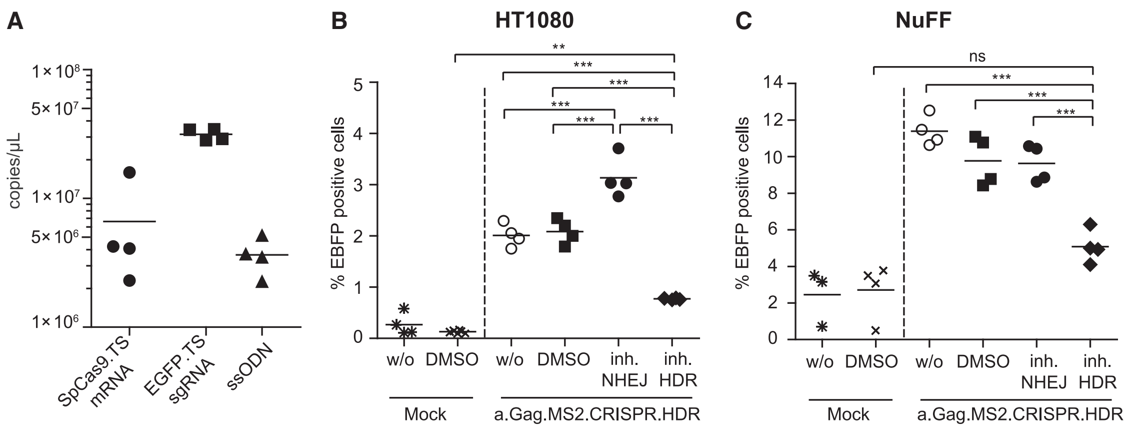

Figure 6. Efficient CRISPR-mediated transition of EGFP to EBFP by a.Gag.MS2.CRISPR.HDR particles

(A) Determined copies of CRISPR RNA and PT-modified ssODN molecules within a.Gag.MS2.CRISPR.HDR supernatants. (B and C) HT1080-based (B) or NuFF-based (C) EGFP reporter cells were transduced with 30 µL (1,980 copies *SpCas9.TS* mRNA/cell) of a.Gag.MS2.CRISPR.HDR supernatants in the absence (w/o) or presence of NHEJ or HDR inhibitors (inh) M3814 or YU238259, respectively. DMSO-treated cells served as solvent controls. The graphs display % EBFP$^+$ cells within treated cultures. Non-treated or DMSO-treated mock cultures indicate the degree of autofluorescence in the EBFP channel. Each data point reflects one independently prepared supernatant (n = 3–4).

EGFP to *EBFP* in a.Gag.MS2.CRISPR.HDR-treated NuFF cells (Figure S5C).

DISCUSSION

Efficient, reversible, and safe delivery of DNA-modifying enzymes, such as TALEN, ZFN, or the CRISPR-Cas9 system, is a prerequisite for gene-editing cell therapies, as stable and high expression levels of these enzymes were shown to be associated with unwanted off-target and/or cytotoxic side effects.[16,59,69] Recently, we developed MLV-based g.Gag.MS2 particles for the transient delivery of CRISPR-Cas9 components into target cells. Redirection of the retrovirus toward the MS2 bacteriophage packaging machinery allowed specific co-packaging of non-retroviral *SpCas9.TS* mRNA and sgRNA.TS transcripts and resulted in an efficient targeted knockout of several genes in various cell types.[16] Although the formerly described technology yielded knockout rates of up to ~80% (Figure 1B), relatively high supernatant volumes were needed (e.g., 50 µL of a 100-fold concentrated supernatant) to obtain robust knockout rates of ≥60%. Therefore, the present study aimed to further improve this technique, as this would save resources and facilitate the transduction of primary cells. We hypothesized that the switch of the MLV-based approach to the phylogenetically more distant alpharetroviral RSV-based vector system might have beneficial effects on the technology, as MLV and RSV evolved in different hosts and therefore interact with different cellular co-factors and/or antiviral restriction factors.[49–54] Strikingly, RSV-based a.Gag.MS2 vector particles revealed significantly higher *RFP657.Tet2* knockout rates in HT1080-based reporter cells (Figure 2C) and allowed efficient transfer of CRISPR-Cas9 components into murine and human primary cells (Figure 5). Furthermore, a.Gag.MS2.CRISPR particles allowed multiplexing (Figure 4), and co-transfection of PT-modified ssODN templates during particle production generated a.Gag.MS2.CRISPR.HDR particles that induced up to 12.5% HDR events in fibroblast-based EGFP/EBFP-switch reporter models (Figure 6).[63]

Overall, the superior performance of a.Gag.MS2 compared with g.Gag.MS2 supernatants may be explained by higher titers. In addition to ~8-fold-higher *SpCas9.TS* mRNA concentrations (Figure 3A), we found ~4-fold elevated levels of structural a.Gag.MS2 precursor proteins (Figure 3C) in a.Gag.MS2-based CRISPR.Tet2 preparations, strongly suggesting higher particle quantities per supernatant volume. Interestingly, and as also shown for g.Gag.MS2.CRISPR particles before[16] as well as here, we detected SpCas9 protein in a.Gag.MS2-based supernatants (Figure 3B). Although SpCas9 protein levels in a.Gag.MS2 preparations clearly fluctuate between supernatant batches, we cannot rule out that transferred SpCas9 protein (either alone or already complexed with a sgRNA) contributed to the measured knockout rates.

During the course of our experiments, we found that the best way to adjust a.Gag.MS2 and g.Gag.MS2 CRISPR.Tet2 particle doses was by normalization based on transferred *SpCas9.TS* mRNA copies per cell (Figure 3). Transduction of HT1080 RFP657.Tet2 reporter cells with serial dilutions of a.Gag.MS2 or g.Gag.MS2 CRISPR.Tet2 supernatants revealed the necessity of applying ~10-fold-more g.Gag.MS2 than a.Gag.MS2 particles to achieve similar *RFP657.Tet2* knockout rates (Figure 3D), a difference that was reflected by the amounts of *SpCas9.TS* mRNA in the respective supernatants (Figure 3A). Interestingly, when retrospectively plotting *RFP657.Tet2* knockout rates against the amount of *SpCas9.TS* mRNA copies that were applied per cell, potency curves of both particle types were similar but did not completely overlap, as a.Gag.MS2.CRISPR.Tet2 particles have ~2-fold-better efficacy (Figure 3E). A potential explanation for this observation is virus- and/or cell-type-related factors (e.g., divergent

expression of antiviral restriction factors and/or supporting co-factors), which either promote or repress the performance of a.Gag.MS2 or g.Gag.MS2 particles, respectively.

The characterization of Gag.MS2.CRISPR.Tet2 supernatants revealed different *SpCas9.TS* mRNA and sgRNA.TS stoichiometries for a.Gag.MS2 and g.Gag.MS2 particles. While a.Gag.MS2.CRISPR.Tet2 supernatants contained on average ~3-fold-more Tet2.TS sgRNA than *SpCas9.TS* mRNA transcripts, the ratio in g.Gag.MS2.CRISPR. Tet2 supernatants was less balanced with an about 65-fold Tet2.TS sgRNA excess over packaged *SpCas9.TS* mRNA (Figure 3A). Comparison of the CRISPR RNA proportions between particle types showed a higher abundance of *SpCas9.TS* mRNA transcripts in a.Gag.MS2- compared with g.Gag.MS2-based particles. In contrast, Tet2.TS sgRNA levels were ~2-fold higher in the less efficient g.Gag.MS2.CRISPR.Tet2 supernatants compared with the more potent a.Gag.MS2.CRISPR.Tet2 supernatants. In relation to ~4-fold reduced Gag.MS2 protein levels in g.Gag.MS2.CRISPR.Tet2 supernatants (Figure 3C), this argues for ~8-fold-more sgRNA.TS copies per particle in g.Gag.MS2 compared with a.Gag.MS2 supernatants. Future studies should determine whether or to what extent the overrepresented sgRNA.TS transcripts in g.Gag.MS2.CRISPR particles influence the particle's life cycle.

As efficient CRISPR-Cas9-mediated gene knockout in target cells strongly depends on the delivery of optimal Cas9/gRNA molar ratios,[70–73] the observed differences in CRISPR RNA packaging might have also influenced the performance of a.Gag.MS2 and g.Gag.MS2 CRISPR.Tet2 particles. Thus, one could envision that the 65-fold excess of Tet2.TS sgRNA transcripts in g.Gag.MS2 particles might be less favorable, which may explain the ~2-fold-better performance of a.Gag.MS2 particles despite the application of adjusted *SpCas9.TS* mRNA quantities (Figure 3E). This non-optimal molar ratio might be due to a higher affinity of g.Gag.MS2 proteins to Tet2.TS sgRNA transcripts, thereby preventing or even inhibiting the incorporation of sufficient *SpCas9.TS* mRNA molecules into assembling particles.

The higher affinity of Gag.MS2 precursor proteins to small sgRNA.TS molecules can be also seen in a.Gag.MS2-based particles, as the addition of every new sgRNA.TS during particle production reduced the incorporation of *SpCas9.TS* mRNA by ~7-fold (compare Figures 3A, 4A, and 4C). This not only argues for an upper limit of incorporation of total sgRNA.TS and *SpCas9.TS* mRNA molecules but also underlines the recruitment of both via the MS2 principle into nascent particles. In contrast, the provision of TS-lacking and DNA-based ssODNs during a.Gag.MS2.CRISPR.HDR particle production did not significantly alter sgRNA.TS/*SpCas9.TS* mRNA ratios (Figure 6A). Noteworthy, in addition to their two mRNA genome copies, wt retroviruses encapsidate several types of host RNA, which are packaged independently of Ψ and which are suggested to contribute to the assembly and structural integrity of retroviral particles.[74] Interestingly, most of these are small RNA-Pol-III-driven non-coding RNA transcripts, which clearly outnumber viral mRNA genomes and randomly packaged host mRNA.[75]

Finally, we demonstrated efficient knockout of endogenous *TP53* or *Trp53* genes in primary human and murine cells by a.Gag.MS2. CRISPR particles (Figure 5). While indel efficiencies in human hepatocytes and NuFFs were comparably high as those obtained in HT1080-based reporter cell lines, gene editing was only low or moderate in hCD34$^+$ HSPCs (6%–36%) and MEFs (18%–31%). Here, it would be interesting to further test other particle doses and/or explore other transduction conditions in combination with small molecules that reversibly interfere with intrinsic cellular immunity.[76–79]

Taken together, the superior performance of a.Gag.MS2.CRISPR supernatants can most likely be explained by enhanced particle titers, more favorable stoichiometries of *SpCas9.TS* mRNA and sgRNA.TS transcripts within particles, and differences in retrovirus biology.

To achieve ~50% knockout efficiency in HT1080-based RFP657.Tet2 reporter cells and highly permissive PHHs and NuFFs, we used ~500–1,000 *SpCas9.TS* mRNA and ~1,000–2,000 sgRNA.TS copies per cell. Comparing this to routinely used physicochemical CRISPR-RNA transfer protocols, which used ~4×10^5–8×10^6 *SpCas9* mRNA copies/cell and ~1×10^8–1×10^9 gRNA copies/cell,[22,72,80] a.Gag.MS2 particles transfer 400- to 16,000-fold-less *SpCas9.TS* mRNA or 50,000- to 1,000,000-fold-less sgRNA.TS transcripts. These data illustrate that spatiotemporal co-delivery of CRISPR-Cas9 components by a.Gag.MS2.CRISPR particles is a cost-effective resource for efficient genome editing of target cells.

As all features necessary for productive cellular entry are retained in a.Gag.MS2 particles, it is very likely that they enter the cell in the same manner as WT retroviruses. This evolved and sophisticated entry strategy not only mediates efficient cellular uptake of a.Gag.MS2 particles but also promotes their efficient endosomal release (and the incorporated RNA cargo) into the cytosol, which is currently the bottleneck of non-viral, particle-based delivery strategies.[25–28] A more detailed comparison of Gag.MS2 particles and other RNA-delivering methods is given in Table S1.

To further improve the a.Gag.MS2-based delivery method, particle-destabilizing agents and/or capsid mutations should be experimentally tested.[81,82] We envision that optimization of mRNA.TS packaging by the introduction of ≥2 MS2 TS hairpins, as for example done in 12- or 24-mer array for mRNA imaging, and/or mRNA stabilizing motifs[83] could further enhance efficiency of this technology.[42] In the case of a.Gag.MS2.CRISPR.HDR particles, higher HDR rates may be achievable by directly tethering the DNA donor to arising particles or, as they randomly package a not inconsiderable amount of SpCas9 protein (Figure 3B), directly to the SpCas9 enzyme.[84] Alternatively, the effectiveness of a.Gag.MS2.CRISPR.HDR particles could be enhanced by combining them with small molecules that not only inhibit the NHEJ DNA repair pathway but also reversibly interfere with the cell cycle.[85] Our virus-based particles, which were pseudotyped with the VSV-G envelope protein, could be combined with novel envelope-based pseudotyping strategies, such as those from measles or Nipah viruses, to allow cell-specific targeting of the

particles to various cell types *in vitro* and *in vivo*.[37,38] In this work, the potency of a.Gag.MS2 particles was demonstrated by short-term delivery of CRISPR-Cas9 components into target cells. However, this technology can be easily extended to applications beyond gene editing and may be exploited in other settings where short-term transgene expression is sufficient to mediate a biological effect. Potential applications are cell homing, cell expansion, or cell differentiation strategies as well as prophylactic or therapeutic tools for vaccination against infectious diseases or cancer. Regarding the multifaceted CRISPR-Cas9 technology, we are confident that a.Gag.MS2-based particles can also be utilized for other genome and chromatin manipulation strategies, including gene regulation, epigenetic editing, and chromatin engineering,[86] all of which contribute to its usefulness in molecular biology and medicine.

MATERIALS AND METHODS

Vector design

The construction of the vectors used in this work is explained in detail in the "Materials and methods" section of the supplemental information.

Cell culture

Human embryonic kidney 293T (293T) cells and human fibroblast HT1080-derived reporter cells were cultured in Dulbecco's modified Eagle's medium (DMEM) with 10% heat-inactivated fetal bovine serum (FBS), 100 U/mL penicillin, 100 μg/mL streptomycin, and 1 mM sodium pyruvate (all from PAN Biotech, Aidenbach, Germany). HT1080 RFP657.Tet2 and HT1080 EGFP reporter cells were additionally cultured in the presence of 1 μg/mL puromycin (InvivoGen, Toulouse, France). Human CXCR4^{+} Jurkat cells[16] stably expressing only the RFP657.Tet2 reporter or the RFP657.Tet2 reporter together with EGFP were cultured in RPMI 1640 medium (PAN Biotech) with the same supplements and concentrations as described above for DMEM-based culture medium together with 0.5 μg/mL puromycin. Primary human NuFF cells (Amsbio, Abingdon, UK) and primary murine C3H (kindly provided by T. Cantz, Hannover Medical School, Germany) and CF1 (Amsbio) embryonic fibroblasts were cultured in DMEM low glucose (PAN Biotech) supplemented with 15% heat-inactivated FBS, 100 U/mL penicillin, 100 μg/mL streptomycin, 2 mM L-glutamine (Biochrom, Berlin, Germany), 1% minimum essential medium (MEM) non-essential amino acids solution (Gibco/Thermo Fisher Scientific, Schwerte, Germany), and 100 μM β-mercaptoethanol. NuFF EGFP reporter cells were cultured in the above-mentioned media supplemented with 1 μg/mL puromycin. PHHs were isolated from liver tissue obtained from patients undergoing partial hepatectomy and according to written informed consent approved by the Ethics Committee of Hannover Medical School (no. 252–2008). Cell isolation was performed using a two-step collagenase perfusion technique as previously reported.[87] PHHs were plated in 6-well culture dishes at 1.5×10^6 cells/well and cultivated in William's medium E supplemented with 1 μM insulin, 1 μM dexamethasone/fortecortin, 100 U/mL penicillin, 100 μg/mL streptomycin, 1 mM sodium pyruvate, 15 mM 4-(2-hydroxyethyl)-1-piperazineethanesulfonic acid (HEPES) buffer, 4 mM L-glutamine, and 5% FBS (all Biochrom). Umbilical-cord-blood-derived hCD34^{+} HSPCs were obtained from the Department of Gynecology and Obstetrics at Hannover Medical School after written informed consent as approved by the Ethics Committtee of Hannover Medical School. Mononuclear cells were separated from diluted cord blood (1:2 with PBS) by means of density gradient centrifugation using Leucosep separation tubes (Greiner Bio-One, Frickenhausen, Germany) and Biocoll (Biochrom) as a separation medium. The isolation of the mononuclear cells was performed according to the manufacturer's instructions. Purification of CD34^{+} HSPC cells was done by magnetic cell separation using the CD34 MicroBead Kit (Miltenyi Biotec, Bergisch Gladbach, Germany) according to the manufacturer's protocol. The CD34^{+} HSPCs were cultured and expanded in serum-free StemSpan medium (STEMCELL Technologies, Cologne, Germany) supplemented with 100 U/mL penicillin, 100 μg/mL streptomycin, 100 ng/mL hSCF, 50 ng/mL hTPO, 100 ng/mL hFLT3 (all PeproTech, Hamburg, Germany), 500 nM StemRegenin1 (STEMCELL Technologies), and 35 nM UM171 (ApexBio, Houston, USA). The hiPSC.RFP657.Tet2 reporter cells are derived from the former described H2E6C hiPSC line[57] and were cultivated feeder free as a monolayer on Geltrex-coated (Gibco/Thermo Fisher Scientific) cell culture dishes with conditioned hiPSC medium (filtered medium from γ-irradiated C3H MEFs) consisting of DMEM/F12 GlutaMAX with 20% knockout serum replacement (both Gibco), 1% non-essential amino acids, 100 U/mL penicillin, 100 μg/mL streptomycin, 2 mM L-glutamine, 100 μM β-mercaptoethanol (Sigma Aldrich, Munich, Germany), 30 ng/mL bFGF (PeproTech), and 0.3 μg/mL puromycin.

Retroviral and Gag.MS2 particle production

Viral supernatants were produced after transient transfection of 293T cells using standard calcium phosphate precipitation.[88] Briefly, the day before transfection 5 to 6×10^6 cells were seeded per 10 cm culture dish (Sarstedt, Nümbrecht, Germany). g.RIT.CRISPR.Tet2 vector particles were generated by co-transfection of 5 μg pSV40enh.RSF91.hU6.sgRNA.Tet2.SpCas9.P2A.EGFP.gPRE[16] gammaretroviral vector expression plasmid with 7 μg pcDNA3.wtMLV.G/P[89] and 2 μg of the VSV-G envelope expression plasmids pMD2.G.[90] For the packaging of lentiviral LIT.SFFV.RFP657.Tet2, LIT.CBX.EFS.RFP657.Tet2, and LIT.CRISPR.Tet2 vector particles, 10 μg lentiviral pLKO5.PPT.SF.RFP657.Tet2.i2.Puro.PRE, pLKO5.PPT.CBX3.EFS.RFP657.Tet2.i2.Puro.PRE, or pLKO5.hU6.sgRNA.Tet2.EFS.SpCas9.P2A.EGFP.PRE[55] vector; 12 μg lentiviral wt Gag-Pol (pcDNA3.wtHIV-1.G/P.4xCTE);[89] 5 μg pRSV.Rev (kindly provided by T. Hope, Northwestern University, Chicago, IL, USA); and 2 μg pMD2.G VSV-G expression plasmids were transfected.

Alpha- or gammaretroviral Gag.MS2.CRISPR particles were generated by transfecting each 2.5 μg of non-retroviral pC1.SpCas9.-P2A.EGFP.TS.gPRE and respective sgRNA.TS expression plasmids together with 5 μg pcDNA3.MLV.CA.pr.MS2CP (g.Gag.MS2) or 3 μg pcDNA3.ASLVco.NC.pr.MS2CP (a.Gag.MS2) and 2 μg pMD2.G VSV-G expression plasmids. For the generation of a.Gag.MS2.CRISPR.HDR particles 0.43 nmol +PT ssODN or −PT ssODN templates were added to the transfection.

In case of Gag.MS2 particle production, respective transfection efficiencies were assessed by flow cytometric analysis of 293T producer cells after the last supernatant harvest. All produced viral supernatants were concentrated 100-fold by ultracentrifugation at either 82,740 × *g* for 2 h or overnight at 13,238 × *g* at 4°C.

Transduction of target cells

The day before transduction, 5×10^4 HT1080 RFP657.Tet2 reporter cells were seeded in a 12-well plate. For the transduction of Jurkat-based reporter cells, 1×10^5 cells were plated per well of a 24-well plate. Indicated amounts of viral supernatants were applied to the cells, and the transduction was assisted by 4 μg/mL protamine sulfate (Sigma Aldrich) and centrifugation at 400 × *g* and 37°C for 1 h. The transduction efficiency was determined via flow cytometry 5 to 6 days post-transduction. hiPSC-based RFP657.Tet2 reporter cells (5×10^4) were transduced with indicated amounts of the respective Gag.MS2.CRISPR.Tet2 supernatants or with LIT.CRISPR.Tet2 particles at an MOI of 2 and 5. Transduction was performed in single-cell suspension in the presence of 4 μg/mL protamine sulfate and 10 μM ROCK-inhibitor Y-27632 (kindly provided by T. Scheper, Leibniz University Hannover, Germany) for 1 h at 37°C and 5% CO_2. Every 10 min, the tubes were flicked to keep the cells in suspension. After the 1 h incubation time, the cells were seeded on Geltrex in conditioned hiPSC medium containing 10 μM ROCK inhibitor for the first 24 h. The medium exchange was accomplished the following day and every other day. Knockout rates were determined 5 days post-transduction via flow cytometry. Primary wt NuFF cells were seeded in a density of 5×10^4 cells/well in a 12-well plate the day before transduction. Transduction was performed with indicated *SpCas9.TS* mRNA vector doses, 8 μg/mL hexadimethrine bromide (Polybrene; Sigma Aldrich), and centrifugation at 400 × *g* and 37°C for 1 h. The PHHs were directly transduced after seeding (1.5×10^6 cells/well) with indicated *SpCas9.TS* mRNA vector doses in the presence of 8 μg/mL Polybrene. For transduction of $hCD34^+$ HSPCs, 1×10^5 cells were plated per well of a 48-well plate and transduction was assisted by addition of 8 μg/mL Polybrene and 1 mg/mL Synperonic F 108 (Sigma-Aldrich). For HDR experiments, 5×10^4 HT1080 EGFP or NuFF EGFP reporter cells were seeded in a 12-well plate. One day later, the cells were transduced in the presence of 4 μg/mL protamine sulfate (HT1080 EGFP) or 8 μg/mL Polybrene (NuFF EGFP) with or without 1 μL/mL DMSO, 5 μM YU238259 (Biomol, Hamburg, Germany), or 1 μM M3814 (Merck, Darmstadt, Germany) followed by centrifugation at 400 × *g* and 37°C for 1 h. HDR efficiency was determined 8 days post-transduction via flow cytometry.

Determination of CRISPR RNA and/or ssODN templates in Gag.MS2 supernatants

To determine the content of *SpCas9.TS* mRNA, respective sgRNA.TS transcripts and/or +PT ssODN templates in Gag.MS2 supernatants, a quantitative real-time PCR was performed. In brief, g.Gag.MS2- and a.Gag.MS2-based particles were produced and 100-fold concentrated, and resulting pellets were resuspended in PBS with 20 mM HEPES. To eliminate potential plasmid contamination, supernatants were treated with 2 U TURBO DNase (Ambion/Thermo Fisher Scientific) for 1 h at 37°C. Subsequently, the RNA was extracted from particles using the QIAGEN RNeasy Micro Kit (QIAGEN, Hilden, Germany) following the manufacturer's protocol with (*SpCas9.TS* mRNA and sgRNA.TS) or without (+PT ssODN) an additional DNase digestion step on the column. To adjust for the potential loss of RNA transcripts or +PT ssODNs during the extraction procedure, DNase-treated supernatants were mixed with a fixed volume of no PRE-encoding RIT.Cre supernatant before RNA extraction. While the amount of +PT ssODNs was directly determined by quantitative real-time PCR after isolation (primers 5′-GGACTTGAAGAAGTCGTGCTG-3′ and 5′-ACCCTCGTGACCACACTG-3′), extracted RNA was reverse transcribed into cDNA prior to quantitative real-time PCR using the QuantiTect Reverse Transcription Kit (QIAGEN). Detection of *SpCas9.TS* cDNA was performed using the PRE-recognizing primer pair 5′-GAGGAGTTGTGGCCCGTGT-3′ and 5′-TGACAGGTGGTGGCAATGCC-3′. The reverse-transcribed sgRNA.TS variants were detected with a common reverse primer 5′-CAAGTTGATAACGGACTAGCCTT-3′ (binding to the sgRNA scaffold) together with a specific forward primer for the different protospacer sequences (5′-GAACAAGCTCTACATCCCGTGT-3′ [Tet2], 5′-GTTCCAGTTTCAGCACATCA-3′ [CXCR4], 5′-GCACTGCACGCCGTAGGTCA-3′ [EGFP], 5′-GACGGAAACCGTAGCTGCCC-3′ [TP53], or 5′-GAGGAGCTCCTGACACTCGGA-3′ [Trp53]). *Cre* cDNA was amplified with primers 5′-AACCATTTGGGCCAGCTAAACA-3′ and 5′-AGAGCCTGTTTTGCACGTTCA-3′. Total amounts of *SpCas9.TS* mRNA, sgRNA.TS, *Cre* mRNA, and +PT ssODN copies were calculated with the help of serial dilutions of individual plasmid or ssODN standards, respectively. The signals for +PT ssODN, *SpCas9.TS*, or sgRNA.TS were corrected with signals obtained for *Cre*.

Determination of SpCas9 protein in Gag.MS2.CRISPR.Tet2 supernatants

The amount of SpCas9 protein in 100-fold concentrated Gag.MS2.CRISPR.Tet2 supernatants was determined via the EpiQuik CRISPR-Cas9 Assay ELISA Kit (Epigentek Group, Farmingdale, NY, USA) following the manufacturer's instructions.

Immunoblot assay of Gag.MS2 supernatants

The content of Gag.MS2 precursor proteins in Gag.MS2 supernatants was analyzed by an immunoblot assay. In brief, 15 μL of each supernatant were mixed with 15 μL of Laemmli buffer and denatured for 5 min at 95°C. Next, 20 μL of each sample was separated by an SDS-PAGE (12.5% separation gel) and transferred to a nitrocellulose membrane (GE Healthcare Life Science, Solingen, Germany). Successful blotting and equal sample loading were controlled by staining the membranes with Ponceau S solution (40% methanol, 15% acidic acid, and 2.5 g/L Ponceau S). Afterward, the membranes were probed with a polyclonal rabbit-anti-enterobacteria phage MS2 coat protein antibody (1:10,000; Merck) in Tris-buffered saline with 0.05% (v/v) Tween and 3% milk powder (both Roth, Karlsruhe, Germany). As a secondary antibody, a goat-anti-rabbit immunoglobulin G (IgG) (1:2,000; Santa Cruz Biotechnology, Heidelberg, Germany) conjugated with horseradish peroxidase was used. Final visualization and quantification were carried out by treating the membrane with

SuperSignal West Pico Chemiluminescent Substrate (Thermo Fisher Scientific) and the FUSION FX imaging system (Vilber Lourmat, Eberhardzell, Germany), respectively.

Statistical analysis

In graph of Figure 4B data are expressed as mean or mean ± standard deviation (SD). For statistical comparison of two groups, we performed an unpaired two-tailed t test. $p > 0.05$ was considered not significant (ns) and $*p \leq 0.05$ was considered significant, $**p \leq 0.01$ very significant, and $***p \leq 0.001$ extremely significant. Significances for Figure 1B were calculated with the one-way analysis of variance (ANOVA) together with the Tukey's post hoc test. For Figure 5A, a two-way ANOVA together with a Bonferroni post-test were performed.

SUPPLEMENTAL INFORMATION

Supplemental information can be found online at https://doi.org/10.1016/j.omtn.2021.12.033.

ACKNOWLEDGMENTS

The authors thank Thomas Neumann, Janne Bretsch, Daniela Lenz, and Daniel Brand for technical support. We are grateful to Dirk Heckl (Department of Pediatrics, University Hospital Halle-Saale, Germany) for providing the LIT.SFFV.RFP657.Tet2 reporter vector construct and the validated wt Tet2 sgRNA. We appreciate that Stefan Horn and Boris Fehse (University Medical Center Hamburg-Eppendorf) kindly provided the CXCR4+ Jurkat cells. We thank Prof. Dr. Thomas J. Hope from Northwestern University, Chicago, IL, USA for providing the pRSV.Rev expression plasmid. Flow cytometric cell sorting was performed at the Central Research Facility Cell Sorting, Hannover Medical School. This work was supported by grants from the German Research Foundation (DFG: Collaborative Research Centers 738/3 [projects C4 and C9] and REBIRTH Cluster of Excellence [EXC 62/1]) and the REBIRTH Center for Translational Regenerative Medicine through the State of Lower Saxony (MWK: ZN3440).

AUTHOR CONTRIBUTIONS

Conceptualization, M.G.; methodology, M.G., Y.B., and A.S.; investigation, Y.B., L.L., D.K., L.N., V.S., and M.G.; resources, A.S., M.-J.K., M.K., P.V.B., J.D.S., F.W.R.V., and D.H.; writing – original draft, Y.B., M.G., and A.S.; writing – review & editing, Y.B., M.G., M.M., L.L., F.W.R.V., and A.S.; visualization, Y.B. and M.G.; supervision, M.G., J.M., M.M., and A.S.; funding acquisition, M.G. and A.S.

DECLARATION OF INTERESTS

The authors declare no competing interests.

REFERENCES

1. Hacein-Bey-Abina, S., Von Kalle, C., Schmidt, M., McCormack, M.P., Wulffraat, N., Leboulch, P., Lim, A., Osborne, C.S., Pawliuk, R., Morillon, E., et al. (2003). LMO2-associated clonal T cell proliferation in two patients after gene therapy for SCID-X1. Science *302*, 415–419.
2. Cartier, N., Hacein-Bey-Abina, S., Bartholomae, C.C., Veres, G., Schmidt, M., Kutschera, I., Vidaud, M., Abel, U., Dal-Cortivo, L., Caccavelli, L., et al. (2009). Hematopoietic stem cell gene therapy with a lentiviral vector in X-linked adrenoleukodystrophy. Science *326*, 818–823.
3. Boyiadzis, M.M., Dhodapkar, M.V., Brentjens, R.J., Kochenderfer, J.N., Neelapu, S.S., Maus, M.V., Porter, D.L., Maloney, D.G., Grupp, S.A., Mackall, C.L., et al. (2018). Chimeric antigen receptor (CAR) T therapies for the treatment of hematologic malignancies: clinical perspective and significance. J. Immunother. Cancer *6*, 137.
4. Mukherjee, S., and Thrasher, A.J. (2013). Gene therapy for PIDs: progress, pitfalls and prospects. Gene *525*, 174–181.
5. Tipanee, J., VandenDriessche, T., and Chuah, M.K. (2017). Transposons: moving forward from preclinical studies to clinical trials. Hum. Gene Ther. *28*, 1087–1104.
6. Hacein-Bey-Abina, S., Garrigue, A., Wang, G.P., Soulier, J., Lim, A., Morillon, E., Clappier, E., Caccavelli, L., Delabesse, E., Beldjord, K., et al. (2008). Insertional oncogenesis in 4 patients after retrovirus-mediated gene therapy of SCID-X1. J. Clin. Invest. *118*, 3132–3142.
7. Ott, M.G., Schmidt, M., Schwarzwaelder, K., Stein, S., Siler, U., Koehl, U., Glimm, H., Kühlcke, K., Schilz, A., Kunkel, H., et al. (2006). Correction of X-linked chronic granulomatous disease by gene therapy, augmented by insertional activation of MDS1-EVI1, PRDM16 or SETBP1. Nat. Med. *12*, 401–409.
8. Braun, C.J., Boztug, K., Paruzynski, A., Witzel, M., Schwarzer, A., Rothe, M., Modlich, U., Beier, R., Göhring, G., Steinemann, D., et al. (2014). Gene therapy for Wiskott-Aldrich syndrome–long-term efficacy and genotoxicity. Sci. Transl. Med. *6*, 227ra33.
9. Kaiser, J. (2021). Gene therapy trials for sickle cell disease halted after two patients develop cancer. https://www.sciencemag.org/news/2021/02/gene-therapy-trials-sickle-cell-disease-halted-after-two-patients-develop-cancer.
10. Maeder, M.L., and Gersbach, C.A. (2016). Genome-editing technologies for gene and cell therapy. Mol. Ther. *24*, 430–446.
11. Nemudryi, A.A., Valetdinova, K.R., Medvedev, S.P., and Zakian, S.M. (2014). TALEN and CRISPR/Cas genome editing systems: tools of discovery. Acta Naturae. *6*, 19–40.
12. Gaj, T., Gersbach, C.A., and Barbas, C.F. (2013). ZFN, TALEN, and CRISPR/Cas-based methods for genome engineering. Trends Biotechnol. *31*, 397–405.
13. Mojica, F.J., Díez-Villaseñor, C., Soria, E., and Juez, G. (2000). Biological significance of a family of regularly spaced repeats in the genomes of Archaea, Bacteria and mitochondria. Mol. Microbiol. *36*, 244–246.
14. Horvath, P., and Barrangou, R. (2010). CRISPR/Cas, the immune system of Bacteria and Archaea. Science *327*, 167–170.
15. Frangoul, H., Altshuler, D., Cappellini, M.D., Chen, Y.-S., Domm, J., Eustace, B.K., Foell, J., de la Fuente, J., Grupp, S., Handgretinger, R., et al. (2021). CRISPR-Cas9 gene editing for sickle cell disease and β-Thalassemia. N. Engl. J. Med. *384*, 252–260.
16. Knopp, Y., Geis, F.K., Heckl, D., Horn, S., Neumann, T., Kuehle, J., Meyer, J., Fehse, B., Baum, C., Morgan, M., et al. (2018). Transient retrovirus-based CRISPR/Cas9 all-in-one particles for efficient, targeted gene knockout. Mol. Ther. Nucleic Acids *13*, 256–274.
17. Kim, S., Kim, D., Cho, S.W., Kim, J., and Kim, J.-S. (2014). Highly efficient RNA-guided genome editing in human cells via delivery of purified Cas9 ribonucleoproteins. Genome Res. *24*, 1012–1019.
18. Gabriel, R., Lombardo, A., Arens, A., Miller, J.C., Genovese, P., Kaeppel, C., Nowrouzi, A., Bartholomae, C.C., Wang, J., Friedman, G., et al. (2011). An unbiased genome-wide analysis of zinc-finger nuclease specificity. Nat. Biotechnol. *29*, 816–823.
19. Geisinger, J.M., and Stearns, T. (2019). CRISPR/Cas9 treatment causes extended TP53-dependent cell cycle arrest in human cells. Nucleic Acids Res. *48*, 9067–9081.
20. Pattanayak, V., Lin, S., Guilinger, J.P., Ma, E., Doudna, J.A., and Liu, D.R. (2013). High-throughput profiling of off-target DNA cleavage reveals RNA-programmed Cas9 nuclease specificity. Nat. Biotechnol. *31*, 839–843.
21. Fu, Y., Foden, J.A., Khayter, C., Maeder, M.L., Reyon, D., Joung, J.K., and Sander, J.D. (2013). High-frequency off-target mutagenesis induced by CRISPR-Cas nucleases in human cells. Nat. Biotechnol. *31*, 822–826.
22. Hendel, A., Bak, R.O., Clark, J.T., Kennedy, A.B., Ryan, D.E., Roy, S., Steinfeld, I., Lunstad, B.D., Kaiser, R.J., Wilkens, A.B., et al. (2015). Chemically modified guide RNAs enhance CRISPR-Cas genome editing in human primary cells. Nat. Biotechnol. *33*, 985–989.

23. De Queiroz, F.M., Sánchez, A., Agarwal, J.R., Stühmer, W., and Pardo, L.A. (2012). Nucleofection induces non-specific changes in the metabolic activity of transfected cells. Mol. Biol. Rep. *39*, 2187–2194.

24. Mo, D., Potter, B.A., Bertrand, C.A., Hildebrand, J.D., Bruns, J.R., and Weisz, O.A. (2010). Nucleofection disrupts tight junction fence function to alter membrane polarity of renal epithelial cells. Am. J. Physiol. Ren. Physiol. *299*, F1178–F1184.

25. Filion, M.C., and Phillips, N.C. (1997). Toxicity and immunomodulatory activity of liposomal vectors formulated with cationic lipids toward immune effector cells. Biochim. Biophys. Acta. *1329*, 345–356.

26. Jones, C.H., Chen, C.-K., Ravikrishnan, A., Rane, S., and Pfeifer, B.A. (2013). Overcoming nonviral gene delivery barriers: perspective and future. Mol. Pharm. *10*, 4082–4098.

27. Gilleron, J., Querbes, W., Zeigerer, A., Borodovsky, A., Marsico, G., Schubert, U., Manygoats, K., Seifert, S., Andree, C., Stöter, M., et al. (2013). Image-based analysis of lipid nanoparticle-mediated siRNA delivery, intracellular trafficking and endosomal escape. Nat. Biotechnol. *31*, 638–646.

28. Pezzoli, D., Chiesa, R., De Nardo, L., and Candiani, G. (2012). We still have a long way to go to effectively deliver genes. J. Appl. Biomater. Funct. Mater. *10*, 82–91.

29. Galla, M., Will, E., Kraunus, J., Chen, L., and Baum, C. (2004). Retroviral pseudotransduction for targeted cell manipulation. Mol. Cell *16*, 309–315.

30. Prel, A., Sensébé, L., and Pagès, J.C. (2013). Influence of untranslated regions on retroviral mRNA transfer and expression. BMC Biotechnol. *13*, 35.

31. Bobis-Wozowicz, S., Galla, M., Alzubi, J., Kuehle, J., Baum, C., Schambach, A., and Cathomen, T. (2014). Non-integrating gamma-retroviral vectors as a versatile tool for transient zinc-finger nuclease delivery. Sci. Rep. *4*, 4656.

32. Mock, U., Riecken, K., Berdien, B., Qasim, W., Chan, E., Cathomen, T., and Fehse, B. (2014). Novel lentiviral vectors with mutated reverse transcriptase for mRNA delivery of TALE nucleases. Sci. Rep. *4*, 6409.

33. Galla, M., Schambach, A., Falk, C.S., Maetzig, T., Kuehle, J., Lange, K., Zychlinski, D., Heinz, N., Brugman, M.H., Göhring, G., et al. (2011). Avoiding cytotoxicity of transposases by dose-controlled mRNA delivery. Nucleic Acids Res. *39*, 7147–7160.

34. Galla, M., Schambach, A., Towers, G.J., and Baum, C. (2008). Cellular restriction of retrovirus particle-mediated mRNA transfer. J. Virol. *82*, 3069–3077.

35. Münch, R.C., Mühlebach, M.D., Schaser, T., Kneissl, S., Jost, C., Plückthun, A., Cichutek, K., and Buchholz, C.J. (2011). DARPins: an efficient targeting domain for lentiviral vectors. Mol. Ther. *19*, 686–693.

36. Kasaraneni, N., Chamoun-Emanuelli, A.M., Wright, G.A., and Chen, Z. (2018). A simple strategy for retargeting lentiviral vectors to desired cell types via a disulfide-bond-forming protein-peptide pair. Sci. Rep. *8*, 10990.

37. Bender, R.R., Muth, A., Schneider, I.C., Friedel, T., Hartmann, J., Plückthun, A., Maisner, A., and Buchholz, C.J. (2016). Receptor-targeted Nipah virus glycoproteins improve cell-type selective gene delivery and reveal a preference for membrane-proximal cell attachment. PLoS Pathog. *12*, e1005641.

38. Buchholz, C.J., Friedel, T., and Büning, H. (2015). Surface-engineered viral vectors for selective and cell type-specific gene delivery. Trends Biotechnol. *33*, 777–790.

39. Lyu, P., Wang, L., and Lu, B. (2020). Virus-like particle mediated CRISPR/Cas9 delivery for efficient and safe genome editing. Life *10*, 366.

40. Prel, A., Caval, V., Gayon, R., Ravassard, P., Duthoit, C., Payen, E., Maouche-Chretien, L., Creneguy, A., Nguyen, T.H., Martin, N., et al. (2015). Highly efficient in vitro and in vivo delivery of functional RNAs using new versatile MS2-chimeric retrovirus-like particles. Mol. Ther. Methods Clin. Dev. *2*, 15039.

41. Lu, B., Javidi-Parsijani, P., Makani, V., Mehraein-Ghomi, F., Sarhan, W.M., Sun, D., Yoo, K.W., Atala, Z.P., Lyu, P., and Atala, A. (2019). Delivering SaCas9 mRNA by lentivirus-like bionanoparticles for transient expression and efficient genome editing. Nucleic Acids Res. *47*, e44.

42. Keryer-Bibens, C., Barreau, C., and Osborne, H.B. (2008). Tethering of proteins to RNAs by bacteriophage proteins. Biol. Cell *100*, 125–138.

43. Peabody, D.S., and Lim, F. (1996). Complementation of RNA binding site mutations in MS2 coat protein heterodimers. Nucleic Acids Res. *24*, 2352–2359.

44. Carey, J., Lowary, P.T., and Uhlenbeck, O.C. (1983). Interaction of R17 coat protein with synthetic variants of its ribonucleic acid binding site. Biochemistry *22*, 4723–4730.

45. Romaniuk, P.J., Lowary, P., Wu, H.N., Stormo, G., and Uhlenbeck, O.C. (1987). RNA binding site of R17 coat protein. Biochemistry *26*, 1563–1568.

46. Suerth, J.D., Maetzig, T., Galla, M., Baum, C., and Schambach, A. (2010). Self-inactivating alpharetroviral vectors with a split-packaging design. J. Virol. *84*, 6626–6635.

47. Mali, P., Yang, L., Esvelt, K.M., Aach, J., Guell, M., DiCarlo, J.E., Norville, J.E., and Church, G.M. (2013). RNA-guided human genome engineering via Cas9. Science *339*, 823–826.

48. Konermann, S., Brigham, M.D., Trevino, A.E., Joung, J., Abudayyeh, O.O., Barcena, C., Hsu, P.D., Habib, N., Gootenberg, J.S., Nishimasu, H., et al. (2015). Genome-scale transcriptional activation by an engineered CRISPR-Cas9 complex. Nature *517*, 583–588.

49. Aiewsakun, P., and Katzourakis, A. (2017). Marine origin of retroviruses in the early Palaeozoic Era. Nat. Commun. *8*, 13954.

50. Weiss, R.A. (2006). The discovery of endogenous retroviruses. Retrovirology *3*, 67.

51. Hayward, A. (2017). Origin of the retroviruses: when, where, and how? Curr. Opin. Virol. *25*, 23–27.

52. Zheng, Y.H., Jeang, K.T., and Tokunaga, K. (2012). Host restriction factors in retroviral infection: promises in virus-host interaction. Retrovirology *9*, 112.

53. Hatziioannou, T., and Bieniasz, P.D. (2011). Antiretroviral restriction factors. Curr. Opin. Virol. *1*, 526–532.

54. Anderson, J.L., and Hope, T.J. (2005). Intracellular trafficking of retroviral vectors: obstacles and advances. Gene Ther. *12*, 1667–1678.

55. Heckl, D., Kowalczyk, M.S., Yudovich, D., Belizaire, R., Puram, R.V., McConkey, M.E., Thielke, A., Aster, J.C., Regev, A., and Ebert, B.L. (2014). Generation of mouse models of myeloid malignancy with combinatorial genetic lesions using CRISPR-Cas9 genome editing. Nat. Biotechnol. *32*, 941–946.

56. Blau, H.M., and Daley, G.Q. (2019). Stem cells in the treatment of disease. N. Engl. J. Med. *380*, 1748–1760.

57. Hoffmann, D., Schott, J.W., Geis, F.K., Lange, L., Müller, F.-J., Lenz, D., Zychlinski, D., Steinemann, D., Morgan, M., Moritz, T., et al. (2017). Detailed comparison of retroviral vectors and promoter configurations for stable and high transgene expression in human induced pluripotent stem cells. Gene Ther. *24*, 298–307.

58. Ackermann, M., Lachmann, N., Hartung, S., Eggenschwiler, R., Pfaff, N., Happle, C., Mucci, A., Göhring, G., Niemann, H., Hansen, G., et al. (2014). Promoter and lineage independent anti-silencing activity of the A2 ubiquitous chromatin opening element for optimized human pluripotent stem cell-based gene therapy. Biomaterials *35*, 1531–1542.

59. Ihry, R.J., Worringer, K.A., Salick, M.R., Frias, E., Ho, D., Theriault, K., Kommineni, S., Chen, J., Sondey, M., Ye, C., et al. (2018). p53 inhibits CRISPR–Cas9 engineering in human pluripotent stem cells. Nat. Med. *24*, 939–946.

60. Hafner, A., Bulyk, M.L., Jambhekar, A., and Lahav, G. (2019). The multiple mechanisms that regulate p53 activity and cell fate. Nat. Rev. Mol. Cell Biol. *20*, 199–210.

61. Mantovani, F., Collavin, L., and Del Sal, G. (2019). Mutant p53 as a guardian of the cancer cell. Cell Death Differ. *26*, 199–212.

62. Brinkman, E.K., Chen, T., Amendola, M., and Van Steensel, B. (2014). Easy quantitative assessment of genome editing by sequence trace decomposition. Nucleic Acids Res. *42*, e168.

63. Flynn, R., Grundmann, A., Renz, P., Hänseler, W., James, W.S., Cowley, S.A., and Moore, M.D. (2015). CRISPR-mediated genotypic and phenotypic correction of a chronic granulomatous disease mutation in human iPS cells. Exp. Hematol. *43*, 838–848.

64. Putney, S.D., Benkovic, S.J., and Schimmel, P.R. (1981). A DNA fragment with an alpha-phosphorothioate nucleotide at one end is asymmetrically blocked from digestion by exonuclease III and can be replicated in vivo. PNAS *78*, 7350–7354.

65. Spitzer, S., and Eckstein, F. (1988). Inhibition of deoxyribonucleases by phosphorothioate groups in oligodeoxyribonucleotides. Nucleic Acids Res. *16*, 11691–11704.

66. Zenke, F.T., Zimmermann, A., Sirrenberg, C., Dahmen, H., Kirkin, V., Pehl, U., Grombacher, T., Wilm, C., Fuchss, T., Amendt, C., et al. (2020). Pharmacologic

inhibitor of DNA-PK, M3814, potentiates radiotherapy and regresses human tumors in mouse models. Mol. Cancer Ther. *19*, 1091–1101.

67. Stachelek, G.C., Peterson-Roth, E., Liu, Y., Fernandez, R.J., Pike, L.R.G., Qian, J.M., Abriola, L., Hoyer, D., Hungerford, W., Merkel, J., et al. (2015). YU238259 is a novel inhibitor of homology-dependent DNA repair that exhibits synthetic lethality and radiosensitization in repair-deficient tumors. Mol. Cancer Res. *13*, 1389–1397.

68. Synthego Performance Analysis (2019). ICE Analysis. v2.0 Synthego. https://www.synthego.com/products/bioinformatics/crispr-analysis.

69. Cornu, T.I., Thibodeau-Beganny, S., Guhl, E., Alwin, S., Eichtinger, M., Joung, J., Cathomen, T., and Cathomen, T. (2008). DNA-binding specificity is a major determinant of the activity and toxicity of zinc-finger nucleases. Mol. Ther. *16*, 352–358.

70. Seki, A., and Rutz, S. (2018). Optimized RNP transfection for highly efficient CRI SPR/Cas9-mediated gene knockout in primary T cells. J. Exp. Med. *215*, 985–997.

71. Liang, X., Potter, J., Kumar, S., Zou, Y., Quintanilla, R., Sridharan, M., Carte, J., Chen, W., Roark, N., Ranganathan, S., et al. (2015). Rapid and highly efficient mammalian cell engineering via Cas9 protein transfection. J. Biotechnol. *208*, 44–53.

72. Kouranova, E., Forbes, K., Zhao, G., Warren, J., Bartels, A., Wu, Y., and Cui, X. (2016). CRISPRs for optimal targeting: delivery of CRISPR components as DNA, RNA, and protein into cultured cells and single-cell embryos. Hum. Gene Ther. *27*, 464–475.

73. Min, Y.L., Li, H., Rodriguez-Caycedo, C., Mireault, A.A., Huang, J., Shelton, J.M., McAnally, J.R., Amoasii, L., Mammen, P.P.A., Bassel-Duby, R., et al. (2019). CRISPR-Cas9 corrects Duchenne muscular dystrophy exon 44 deletion mutations in mice and human cells. Sci. Adv. *5*, eaav4324.

74. Muriaux, D., Mirro, J., Harvin, D., and Rein, A. (2001). RNA is a structural element in retrovirus particles. PNAS *98*, 5246–5251.

75. Telesnitsky, A., and Wolin, S.L. (2016). The host RNAs in retroviral particles. Viruses *8*, 235.

76. Schott, J.W., León-Rico, D., Ferreira, C.B., Buckland, K.F., Santilli, G., Armant, M.A., Schambach, A., Cavazza, A., and Thrasher, A.J. (2019). Enhancing lentiviral and alpharetroviral transduction of human hematopoietic stem cells for clinical application. Mol. Ther. Methods Clin. Dev. *14*, 134–147.

77. Petrillo, C., Cesana, D., Piras, F., Bartolaccini, S., Naldini, L., Montini, E., and Kajaste-Rudnitski, A. (2015). Cyclosporin A and rapamycin relieve distinct lentiviral restriction blocks in hematopoietic stem and progenitor cells. Mol. Ther. *23*, 352–362.

78. Petrillo, C., Thorne, L.G., Unali, G., Schiroli, G., Giordano, A.M.S., Piras, F., Cuccovillo, I., Petit, S.J., Ahsan, F., Noursadeghi, M., et al. (2018). Cyclosporine H overcomes innate immune restrictions to improve lentiviral transduction and gene editing in human hematopoietic stem cells. Cell Stem Cell *23*, 820–832.e9.

79. Geis, F.K., Galla, M., Hoffmann, D., Kuehle, J., Zychlinski, D., Maetzig, T., Schott, J.W., Schwarzer, A., Goffinet, C., Goff, S.P., et al. (2017). Potent and reversible lentiviral vector restriction in murine induced pluripotent stem cells. Retrovirology *14*, 34.

80. Kelley, M.L., Strezoska, Ž., He, K., Vermeulen, A., and Smith, A.van B. (2016). Versatility of chemically synthesized guide RNAs for CRISPR-Cas9 genome editing. J. Biotechnol. *233*, 74–83.

81. Shi, J., Zhou, J., Shah, V.B., Aiken, C., and Whitby, K. (2011). Small-molecule inhibition of human immunodeficiency virus type 1 infection by virus capsid destabilization. J. Virol. *85*, 542–549.

82. Cairns, T.M., and Craven, R.C. (2001). Viral DNA synthesis defects in assembly-competent Rous sarcoma virus CA mutants. J. Virol. *75*, 242–250.

83. Geisberg, J.V., Moqtaderi, Z., Fan, X., Ozsolak, F., and Struhl, K. (2014). Global analysis of mRNA isoform half-lives reveals stabilizing and destabilizing elements in yeast. Cell *156*, 812–824.

84. Aird, E.J., Lovendahl, K.N., St Martin, A., Harris, R.S., and Gordon, W.R. (2018). Increasing Cas9-mediated homology-directed repair efficiency through covalent tethering of DNA repair template. Commun. Biol. *1*, 54.

85. Lin, S., Staahl, B.T., Alla, R.K., and Doudna, J.A. (2014). Enhanced homology-directed human genome engineering by controlled timing of CRISPR/Cas9 delivery. Elife *3*, e04766.

86. Adli, M. (2018). The CRISPR tool kit for genome editing and beyond. Nat. Commun. *9*, 1911.

87. Kleine, M., Riemer, M., Krech, T., DeTemple, D., Jäger, M.D., Lehner, F., Manns, M.P., Klempnauer, J., Borlak, J., Bektas, H., et al. (2014). Explanted diseased livers - a possible source of metabolic competent primary human hepatocytes. PLoS One *9*, e101386.

88. Galla, M., Schambach, A., and Baum, C. (2013). Retrovirus-based mRNA transfer for transient cell manipulation. Methods Mol. Biol. *969*, 139–161.

89. Schambach, A., Bohne, J., Chandra, S., Will, E., Margison, G.P., Williams, D.A., and Baum, C. (2006). Equal potency of gammaretroviral and lentiviral SIN vectors for expression of O6-methylguanine-DNA methyltransferase in hematopoietic cells. Mol. Ther. *13*, 391–400.

90. Yang, Y., Vanin, E.F., Whitt, M.A., Fornerod, M., Zwart, R., Schneiderman, R.D., Grosveld, G., and Nienhuis, A.W. (1995). Inducible, high-level production of infectious murine leukemia retroviral vector particles pseudotyped with vesicular stomatitis virus G envelope protein. Hum. Gene Ther. *6*, 1203–1213.

OMTN, Volume *27*

Supplemental information

Improved alpharetrovirus-based Gag.MS2 particles for efficient and transient delivery of CRISPR-Cas9 into target cells

Yvonne Baron, Johanna Sens, Lucas Lange, Larissa Nassauer, Denise Klatt, Dirk Hoffmann, Marc-Jens Kleppa, Philippe Vollmer Barbosa, Maximilian Keisker, Viviane Steinberg, Julia D. Suerth, Florian W.R. Vondran, Johann Meyer, Michael Morgan, Axel Schambach, and Melanie Galla

Supplemental Material and Methods

Cloning strategies

Lentiviral CRISPR/Cas9 all-in-one vector plasmids pLKO5.hU6.sgRNA.Tet2.PPT.EFS.SpCas9.P2A. EGFP.gPRE (HIV-1-based LIT.CRISPR.Tet2)[1] as well as the LIT.SFFV.RFP657.Tet2 reporter construct (pLKO5.PPT.SF.RFP657.Tet2.i2.Puro.PRE)[1] were designed and generated in former studies.

To enable silencing resistant expression of the *RFP657.Tet2* reporter cassette in hiPSC, we replaced the SFFV promoter in the LIT.SFFV.RFP657.Tet2 reporter depicted in Figure S1A[1] by a promoter configuration which was validated to most efficiently inhibit epigenetic silencing in hiPSC.[2] The promoter configuration consisted of the minimal ubiquitous chromatin opening element CBX3[3] juxtaposed to the elongation factor 1 alpha short (EFS) promoter and was inserted as an XhoI/AgeI fragment into the LIT.SFFV.RFP657.Tet2 reporter plasmid. The resulting lentiviral reporter plasmid was named LIT.CBX.EFS.RFP657.Tet2 (pLKO5.PPT.CBX3.EFS.RFP657.Tet2.i2.Puro.PRE).

The cloning strategies for g.CA.pr.MS2CP (pcDNA3.MLV.Gag.CA.pr.MS2CP), g.Pol, *SpCas9.TS*, and Tet2.TS, TP53.TS, Trp53.TS or CXCR4.TS sgRNA expression constructs are described elsewhere.[4]

For EGFP.TS.inc sgRNA expression a non-retroviral expression plasmid was generated (pCMV.DsRedexp.hU6.sgRNA.EGFP2.TS.inc.pA) by introducing phosphorylated and annealed oligonucleotides 5'-CACCGCACTGCACGCCGTAGGTCA-3' and 5'-AAACTGACCTACGGCGTGCA GTGC-3' into the BsmBI site of pCMV.DsRedexp.hU6.BsmBI-Stuffer.TS.inc.[4]

For the generation of pcDNA3.ASLV.Gag.co.NC.pr.MS2 (a.Gag.MS2), we first generated alpharetroviral Gag expression plasmids with codon-optimized subdomains (MA, p2, p10, CA and NC) with or without the retroviral protease site adjacent to NC. The codon-optimized incomplete a.Gag fragment lacking the PR subdomain was amplified with primers 5'-CGTGAGATCTGAATTCGC CACCATG-3' and 5'-CCATACCGGTGCTCACGGCCAGGCTCACGGCAGGCTC-3' from the codon-optimized alpharetroviral wt Gag-Pol expression plasmid (pcDNA3.wtAlpha.G/P.co).[5] Subsequently, the PCR fragments were inserted into the EcoRI/AgeI digested pcDNA3.ASLV.Gag.NC-GFP backbone (Gag part is not codon-optimized) to generate codon-optimized (Gag part only) pcDNA3.ASLV.Gag.co.NC.pr.EGFP. Finally, the *EGFP* transgene was replaced by the *MS2CP* heterodimer sequence[4] by restriction enzymes AgeI and NotI.

Genetic detection of HDR events

For genetic analysis of a.Gag.MS2.CRISPR.HDR-induced HDR events in HT1080-based EGFP reporter cells, selected cell samples (treated or untreated) from Figure 6B were sorted for EBFP positive cells 13 d after transduction. gDNA was isolated from these cell populations with the QIAamp DNA Blood Mini Kit (QIAGEN) following the manufacturer's instructions. Subsequently, the respective locus of the *EGFP/EBFP* gene was amplified by primers 5'-ATGGTGAGCAAGGGCGAGGAG-3' and 5'-CTTGTAC AGCTCGTCCATGCCG-3' using the Phire Tissue Direct PCR Master Mix (Thermo Fisher Scientific). Finally, the obtained PCR fragment was sequenced with the primer 5'-TTGAAGTTCACCTTGATG CCG-3' and the sequencing results were analyzed by the online ICE v2 CRISPR Analysis Tool (Synthego).[6] For the molecular conformation of HDR events in NuFF-based

EGFP reporter cells, selected bulk cultures from Figure 6C were harvested 3 d after transduction according to the instructions of the Thermo Scientific Phire Tissue Direct PCR Master Mix kit. The *EGFP/EBFP* locus was amplified as described above and the resulting amplicons were subcloned into a shuttle vector. After transformation into XL1-blue *Escherichia coli* bacteria (Agilent, Waldbronn, Germany), the plasmid DNA of bacterial colonies was extracted and analyzed for positive HDR events via sequencing and the online ICE v2 CRISPR Analysis Tool.

Table S1

Table S1: Comparison of Gag.MS2 particles and non-viral RNA delivery methods: Advantages and disadvantages of respective technologies.

RNA Delivery Method	Advantage(s)	Disadvantage(s)	Exemplary References
Naked RNA delivery	Simple; *in vivo* application; easy upscaling	Ineffective cellular uptake; prone to nuclease degradation; can be immunogenic; fast systemic clearance	[7–12]
Electroporation	Highly efficient; high reproducibility; easy upscaling	Voltage-induced cytotoxicity; no cell targeting; mainly for *ex-vivo* applications	[12–18]
Lipid-based nanocarriers	Moderate efficiency; low to moderate cytotoxicity; protection from nucleases; *in vivo* application; low immunogenicity	Inefficient endosomal escape; concerns about *in vivo* biodegradability; specific tissue targeting is challenging; cell type- and composition-dependent cytotoxicity; limitations in fabrication reproducibility	[12,17,19–23]
Polymer-based nanocarriers	Moderate efficiency; low to moderate cytotoxicity; protection from nuclease; *in vivo* application; capacity for cell-specific targeting; low immunogenicity; facile synthesis	Inefficient endosomal escape; cell type- and composition-dependent cytotoxicity	[12,17,20,23–26]
Direct Ligand-RNA conjugates	Limited cytotoxicity; *in vivo* application; cell-specific targeting	High RNA doses required; dependent on RNA-stabilizing modifications; only shown for small regulatory RNA	[11,27–30]
Retrovirus-based Gag.MS2 particles	Highly efficient; delivery of non-retroviral RNA; low immunogenicity; efficient endosomal escape; capacity for cell-specific targeting and *in vivo* application; relatively well-tolerated procedure	Efficiency is dependent on cellular co-factor and/or antiviral restriction factor expression; producer cells needed for particle generation	[4,31–40]<br>this paper

Figure S1

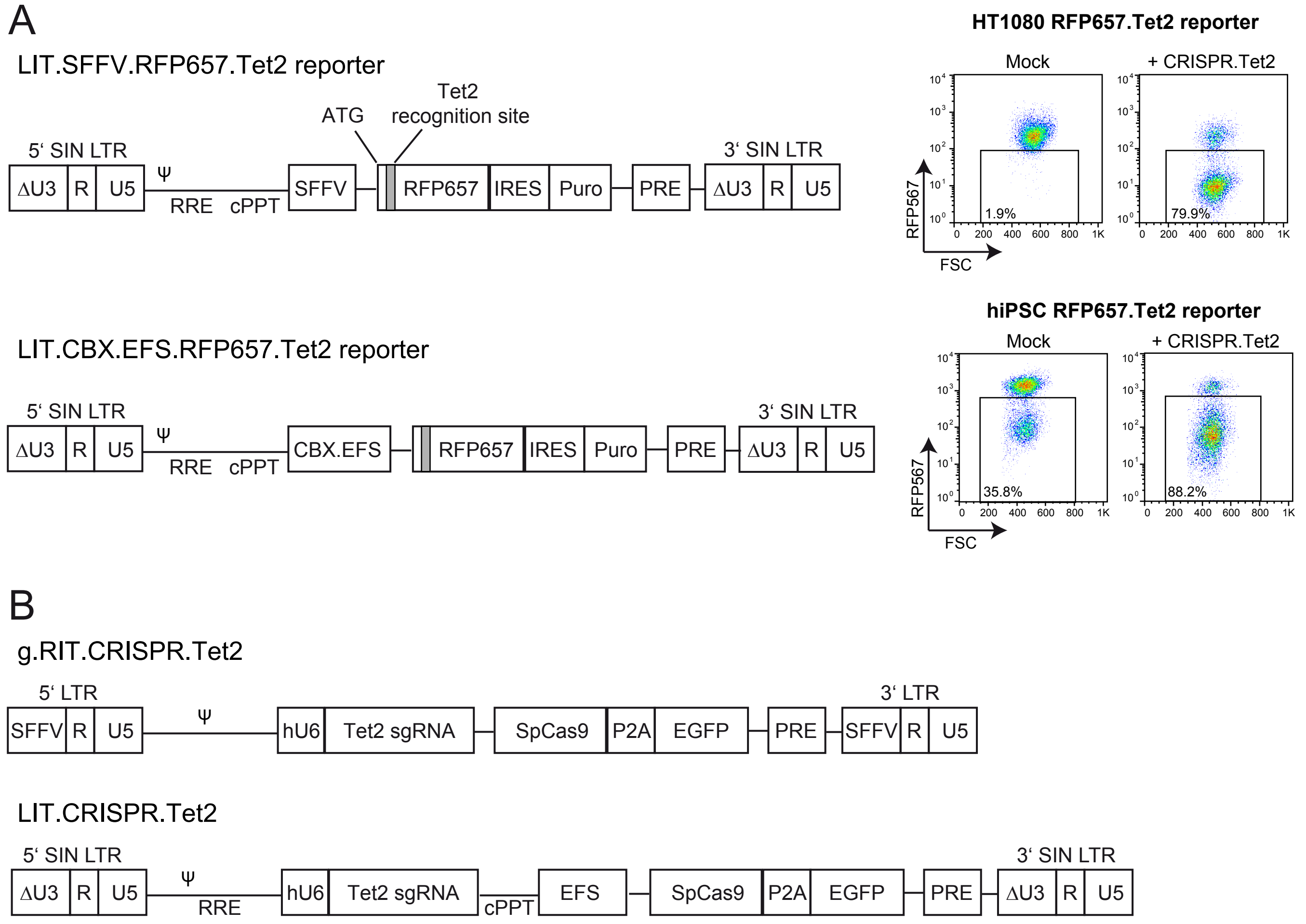

Figure S1. Schematic illustration of integrating retroviral vectors used for CRISPR/Cas9 experiments in this study. (**A**) Lentiviral integrating transfer (LIT) vector encoding the RFP657.Tet2 reporter cassette. The far-red *RFP657* gene is expressed from the spleen focus forming virus (SFFV) promoter (top) or the silencing resistant CBX3.EFS promoter (bottom). The latter consists of a minimal ubiquitous chromatin opening element (CBX3) fused to the elongation factor 1 alpha short (EFS) promoter. The *RFP657* gene contains the sgRNA recognition site (including native AGG PAM) for the mouse *Tet2* gene downstream of its start codon (ATG). *RFP657.Tet2* is expressed together with the puromycin resistance gene (Puro) via the internal ribosomal entry site (IRES) of the endomyocarditis virus. Representative FACS plots of non-transduced (Mock) or a.Gag.MS2.CRISPR.Tet2-transduced HT1080- or hiPSC-based RFP657.Tet2 reporters are shown on the right. (**B**) LIT and gammaretroviral transfer (g.RIT) CRISPR/Cas9 all-in-one vectors targeting mouse *Tet2*. In contrast to the long terminal repeat (LTR)-driven g.RIT.CRISPR.Tet2 vector, respective LIT vectors display self-inactivating (SIN) design. sgRNA expression is driven by the human Pol III promoter U6 (hU6), and the *Streptococcus pyogenes* Cas9 (SpCas9) is either co-expressed with green-fluorescent EGFP or the red-fluorescent dTomato protein via 2A self-cleavage sites from porcine teschovirus-1 (P2A) or thosea asigna virus (T2A). PRE: post-transcriptional regulatory element from woodchuck hepatitis virus; Ψ: retroviral packaging signal; RRE: Rev responsive element; cPPT: central polypurine tract.

Figure S2

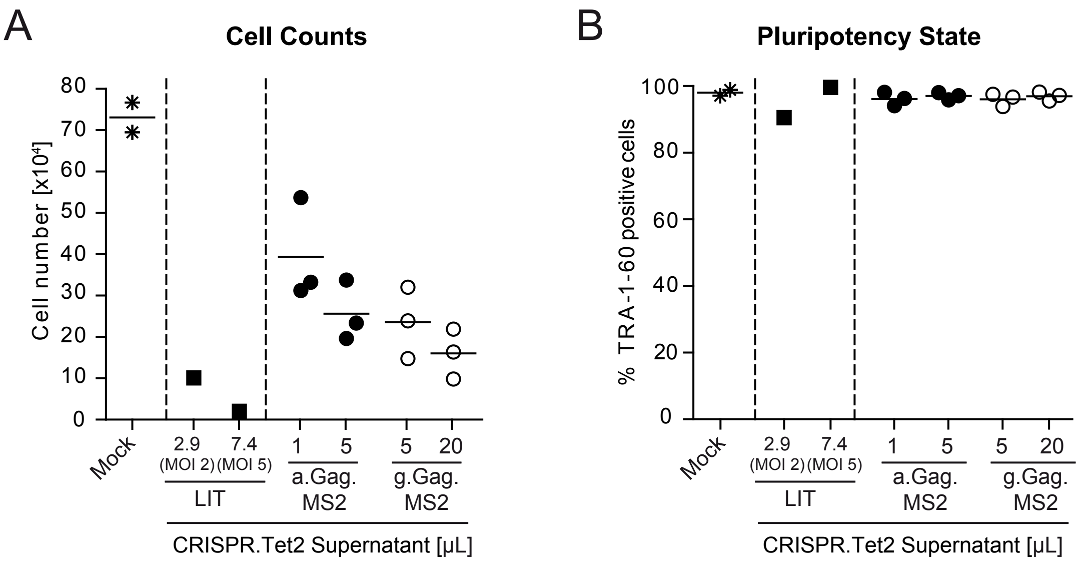

Figure S2. Reduced cytotoxicity in hiPSC that were treated with Gag.MS2-based CRISPR.Tet2 particles. Absolute cell numbers (**A**) and the respective pluripotency state (**B**) of transduced hiPSC-based RFP657.Tet2 reporter cultures from Figure 2D are shown. Integrating LIT.CRISPR.Tet2 particles served as control. Each data point represents one individually generated supernatant. TRA-1-60: pluripotency marker for hiPSC.

Figure S3

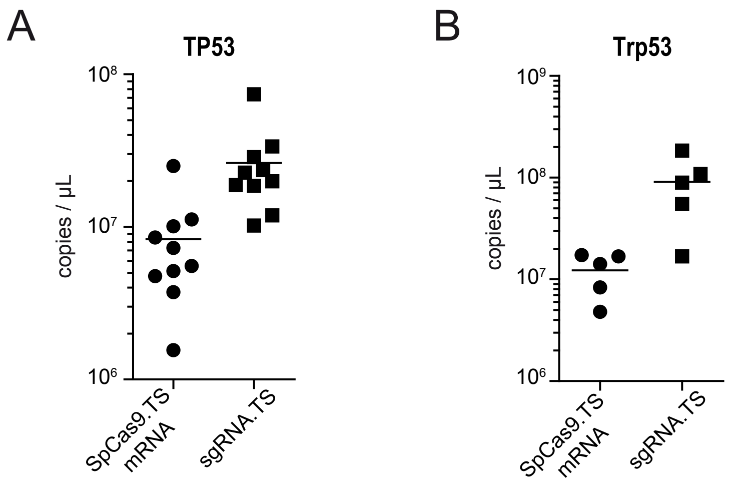

Figure S3. Quantification of SpCas9.TS mRNA and sgRNA.TS transcripts in a.Gag.MS2.CRISPR supernatants that were used for the transduction of primary cells (Figure 5). The concentrations of CRISPR RNA transcripts in supernatants containing particles targeting either human *TP53* (**A**) or murine *Trp53* (**B**) genes are shown.

Figure S4

A

Single-Stranded HDR Donor DNA Oligonucleotide (ssODN)

CCTTCGGGCATGGCGGACTTGAAGAAGTCGTGCTGCTTCATGTGGTCTGGGTAGCGGCTG
AAGCACTGCACGCCGTGGCTCAGTGTGGTCACGAGGGTGGGCCAGGGCACGGGCAGCTTG

B

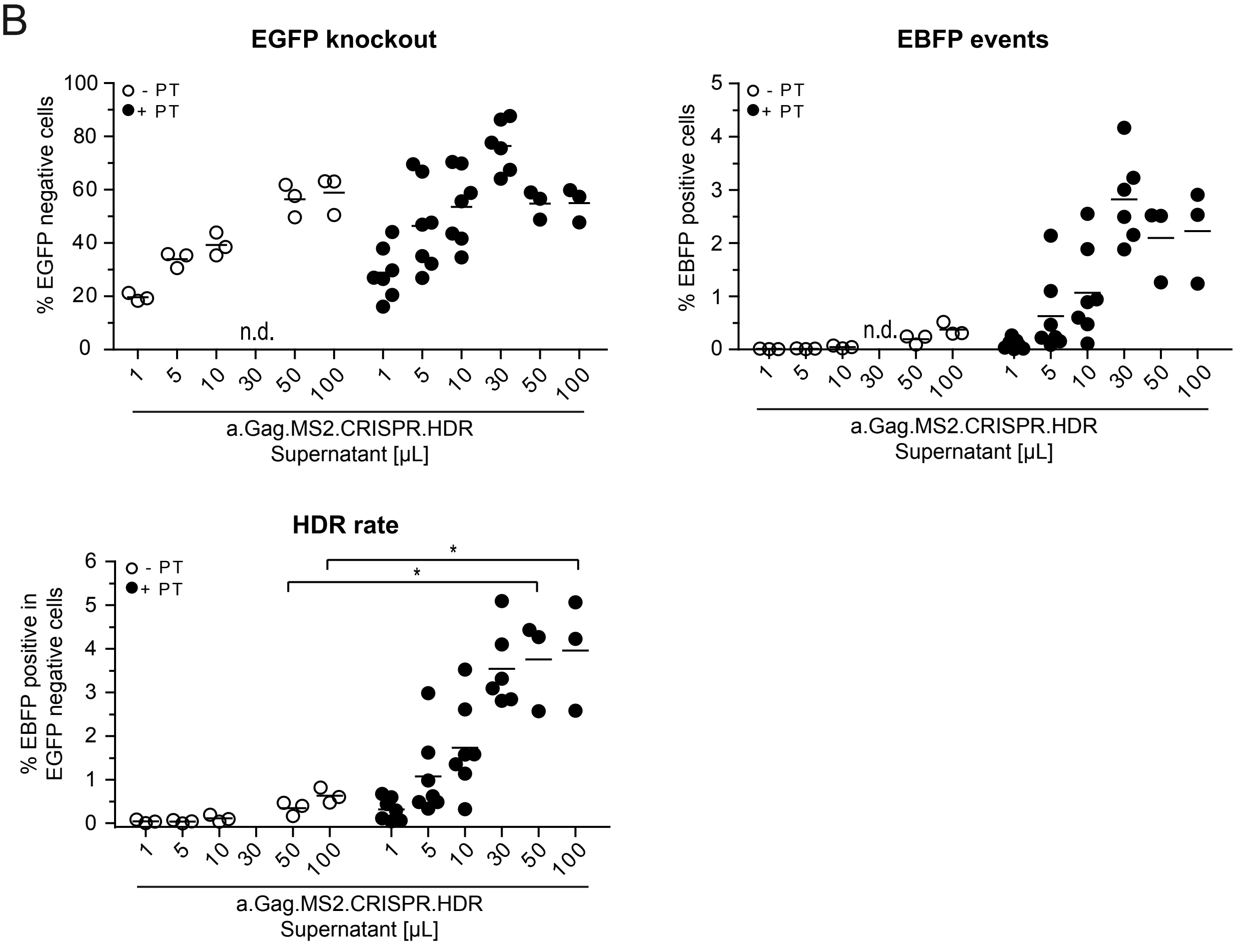

Figure S4. Efficient CRISPR/Cas9-induced HDR by a.Gag.MS2.CRISPR.HDR particles. Proof of concept was shown with the help of the recently published EGFP to EBFP transition model.[41] (**A**) Respective single-stranded DNA oligonucleotide (ssODN) donor sequence. The 120 bp long ssODN used in B, Figure 6 and Figure S5 contains two point mutations (in red) conferring the amino acid changes T66S and Y67H thereby switching EGFP to EBFP expression. The mutations are flanked by asymmetric homology arms (in black). An additional silent point mutation (in blue) within PAM should avoid CRISPR/Cas9-mediated cleavage of the HDR donor template. The black line indicates the EGFP.TS sgRNA target sequence with the exception of the 2 point mutations in red. The donor was purchased with and without phosphorothioate (+PT or -PT, respectively) modifications. (**B**) Co-transfected +PT ssODN during particle production enhanced HDR. +PT or -PT ssODN donors (0.43 nmol) were co-transfected during a.Gag.MS2.CRISPR.HDR particle production. HT1080-based EGFP HDR reporter cells were transduced with indicated supernatant volumes and were analyzed for EGFP$^-$ (upper left graph) and EBFP$^+$ positive cells (upper right graph) via flow cytometry. The calculated HDR rate (%EBFP$^+$ within EGFP$^-$ cells) is depicted at the lower left. The results of three to seven independent supernatants are depicted (N=3-7). n.d.: not determined.

Figure S5

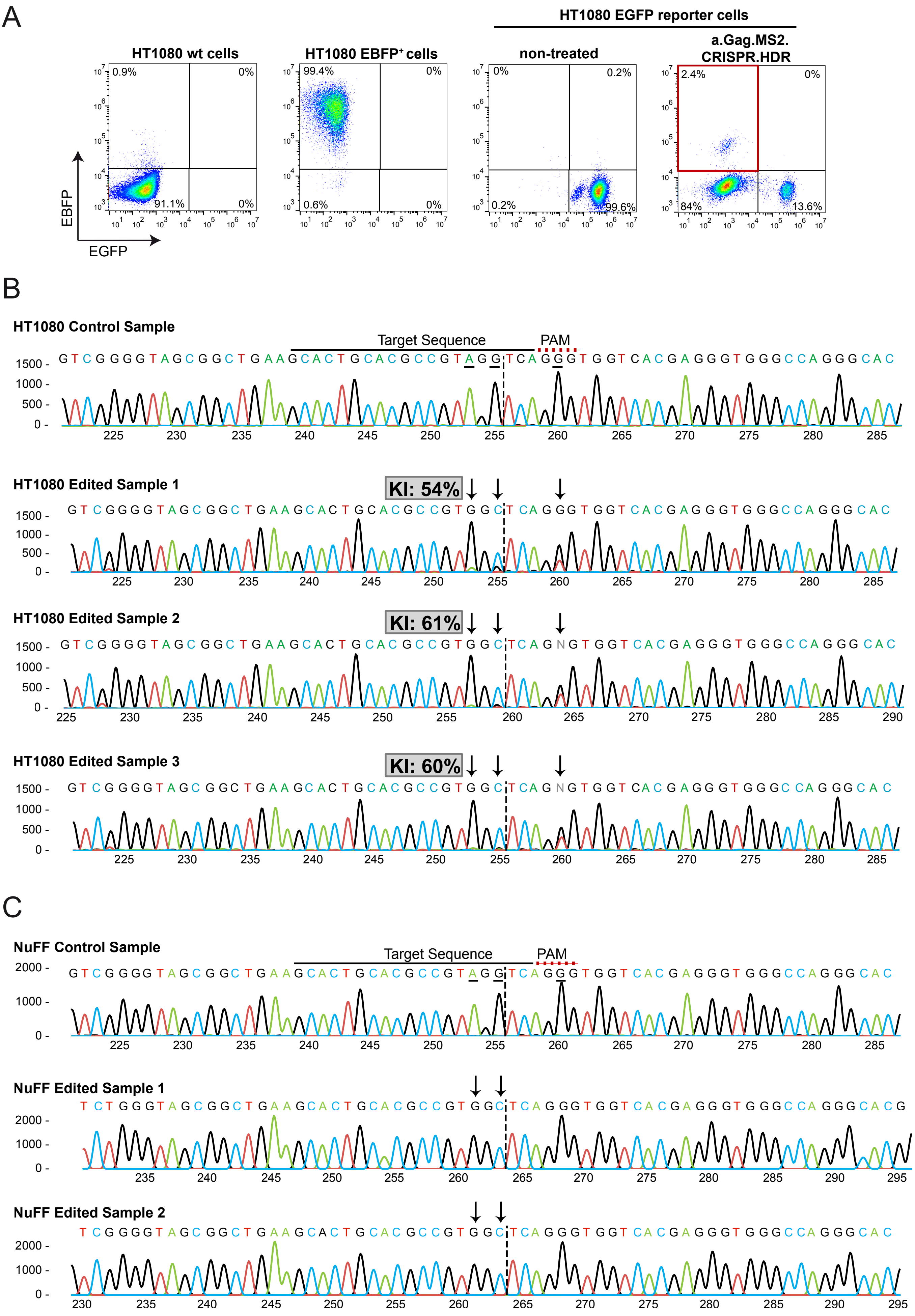
A
HT1080 EGFP reporter cells
HT1080 wt cells
HT1080 EBFP+ cells
non-treated
a.Gag.MS2. CRISPR.HDR
0.9%
0%
91.1%
0%
99.4%
0%
0.6%
0%
0%
0.2%
0.2%
99.6%
2.4%
0%
84%
13.6%
EBFP
EGFP
B
HT1080 Control Sample
Target Sequence
PAM
HT1080 Edited Sample 1
KI: 54%
HT1080 Edited Sample 2
KI: 61%
HT1080 Edited Sample 3
KI: 60%
C
NuFF Control Sample
Target Sequence
PAM
NuFF Edited Sample 1
NuFF Edited Sample 2

Figure S5. Molecular analysis of HDR events in HT1080-based and NuFF-based EGFP reporter cells. (**A**) Representative flow cytometry plots showing the gating strategy for the sorting of edited $EBFP^+/EGFP^-$ cells (red box) 13 d after a.Gag.MS2.CRISPR.HDR treatment. (**B**) Knockin (KI) rate analyses of sorted $EBFP^+/EGFP^-$ cells. The genomic DNA of sorted cells from 3 independent experiments (Figure 6B) was extracted and sequenced. Resulting sequences were subjected to the ICE analysis tool (Synthego).[6] The „Edited Sample 1" is derived from cultures that were transduced without inhibitor. „Edited Samples 2 & 3" are derived from cells that were treated with a.Gag.MS2.CRISPR.HDR particles in the presence of the NHEJ inhibitor M3814. (**C**) Molecular analysis of HDR events in a.Gag.MS2.CRISPR.HDR-treated NuFF.EGFP reporter cells from Figure 6C. The respective site within *EGFP* was amplified and the amplicon was subcloned into a shuttle vector. After transformation, bacterial clones were screened for positive HDR events. Sequencing results were analyzed with the help of the ICE analysis tool. The switch from EGFP to EBFP in bulk cultures of two individual transductions (Edited Sample 1 and 2) is shown.

REFERENCES

1. Heckl, D., Kowalczyk, M.S., Yudovich, D., Belizaire, R., Puram, R. V, McConkey, M.E., Thielke, A., Aster, J.C., Regev, A., and Ebert, B.L. (2014). Generation of mouse models of myeloid malignancy with combinatorial genetic lesions using CRISPR-Cas9 genome editing. Nat. Biotechnol. *32*, 941–946.

2. Hoffmann, D., Schott, J.W., Geis, F.K., Lange, L., Müller, F.-J., Lenz, D., Zychlinski, D., Steinemann, D., Morgan, M., Moritz, T., et al. (2017). Detailed comparison of retroviral vectors and promoter configurations for stable and high transgene expression in human induced pluripotent stem cells. Gene Ther. *24*, 298–307.

3. Müller-Kuller, U., Ackermann, M., Kolodziej, S., Brendel, C., Fritsch, J., Lachmann, N., Kunkel, H., Lausen, J., Schambach, A., Moritz, T., et al. (2015). A minimal ubiquitous chromatin opening element (UCOE) effectively prevents silencing of juxtaposed heterologous promoters by epigenetic remodeling in multipotent and pluripotent stem cells. Nucleic Acids Res. *43*, 1577–1592.

4. Knopp, Y., Geis, F.K., Heckl, D., Horn, S., Neumann, T., Kuehle, J., Meyer, J., Fehse, B., Baum, C., Morgan, M., et al. (2018). Transient Retrovirus-Based CRISPR/Cas9 All-in-One Particles for Efficient, Targeted Gene Knockout. Mol. Ther. Nucleic Acids *13*, 256–274.

5. Suerth, J.D., Maetzig, T., Galla, M., Baum, C., and Schambach, A. (2010). Self-inactivating alpharetroviral vectors with a split-packaging design. J. Virol. *84*, 6626–6635.

6. Synthego (2019). Synthego Performance Analysis, ICE Analysis. v2.0. [accessed 2021/02/01].

7. Houseley, J., and Tollervey, D. (2009). The Many Pathways of RNA Degradation. Cell *136*, 763–776.

8. Tsui, N.B.Y., Ng, E.K.O., and Lo, Y.M.D. (2002). Stability of endogenous and added RNA in blood specimens, serum, and plasma. Clin. Chem. *48*, 1647–1653.

9. Wojtczak, B.A., Sikorski, P.J., Fac-Dabrowska, K., Nowicka, A., Warminski, M., Kubacka, D., Nowak, E., Nowotny, M., Kowalska, J., and Jemielity, J. (2018). 5′-Phosphorothiolate Dinucleotide Cap Analogues: Reagents for Messenger RNA Modification and Potent Small-Molecular Inhibitors of Decapping Enzymes. J. Am. Chem. Soc. *140*, 5987–5999.

10. Li, B., Luo, X., and Dong, Y. (2016). Effects of Chemically Modified Messenger RNA on Protein Expression. Bioconjug. Chem. *27*, 849–853.

11. Kaczmarek, J.C., Kowalski, P.S., and Anderson, D.G. (2017). Advances in the delivery of RNA therapeutics: From concept to clinical reality. Genome Med. *9*, 60.

12. Ramamoorth, M., and Narvekar, A. (2015). Non viral vectors in gene therapy - An overview. J. Clin. Diagn Res. *9*, GE01–GE06.

13. Mo, D., Potter, B.A., Bertrand, C.A., Hildebrand, J.D., Bruns, J.R., and Weisz, O.A. (2010). Nucleofection disrupts tight junction fence function to alter membrane polarity of renal epithelial cells. Am. J. Physiol. Renal Physiol. *299*, F1178-1184.

14. De Queiroz, F.M., Sánchez, A., Agarwal, J.R., Stühmer, W., and Pardo, L.A. (2012). Nucleofection induces non-specific changes in the metabolic activity of transfected cells. Mol. Biol. Rep. *39*, 2187–2194.

15. Van Driessche, A., Ponsaerts, P., Van Bockstaele, D.R., Van Tendeloo, V.F.I., and Berneman, Z.N. (2005). Messenger RNA electroporation: An efficient tool in immunotherapy and stem cell research. Folia Histochem. Cytobiol. *43*, 213–216.

16. Johansson, D.X., Ljungberg, K., Kakoulidou, M., and Liljeström, P. (2012). Intradermal Electroporation of Naked Replicon RNA Elicits Strong Immune Responses. PLoS One *7*, e29732.

17. Bono, N., Ponti, F., Mantovani, D., and Candiani, G. (2020). Non-viral in vitro gene delivery: It is now time to set the bar! Pharmaceutics *12*, 183.

18. Rols, M.-P. (2017). Parameters Affecting Cell Viability Following Electroporation In Vitro. In Handbook of Electroporation, pp. 1449–1465.

19. Kowalski, P.S., Rudra, A., Miao, L., and Anderson, D.G. (2019). Delivering the Messenger: Advances in Technologies for Therapeutic mRNA Delivery. Mol. Ther. *27*, 710–728.

20. Pezzoli, D., Chiesa, R., De Nardo, L., and Candiani, G. (2012). We still have a long way to go to effectively deliver genes! J. Appl. Biomater. Funct. Mater. *10*, 82–91.

21. Xue, H., Guo, P., Wen, W.-C., and Wong, H. (2015). Lipid-Based Nanocarriers for RNA Delivery. Curr. Pharm. Des. *21*, 3140–3147.

22. Moss, K.H., Popova, P., Hadrup, S.R., Astakhova, K., and Taskova, M. (2019). Lipid Nanoparticles for Delivery of Therapeutic RNA Oligonucleotides. Mol. Pharm. *16*, 2265–2277.

23. Jones, C.H., Chen, C.-K., Ravikrishnan, A., Rane, S., and Pfeifer, B.A. (2013). Overcoming Nonviral Gene Delivery Barriers: Perspective and Future. Mol. Pharm. *10*, 4082–4098.

24. Cai, J., Yue, Y., Rui, D., Zhang, Y., Liu, S., and Wu, C. (2011). Effect of chain length on cytotoxicity and endocytosis of cationic polymers. Macromolecules *44*, 2050–2057.

25. Pack, D.W., Hoffman, A.S., Pun, S., and Stayton, P.S. (2005). Design and development of polymers for gene delivery. Nat. Rev. Drug Discov. *4*, 581–593.

26. Rai, R., Alwani, S., and Badea, I. (2019). Polymeric nanoparticles in gene therapy: New avenues of design and optimization for delivery applications. Polymers (Basel). *11*, 745.

27. Nair, J.K., Willoughby, J.L.S., Chan, A., Charisse, K., Alam, M.R., Wang, Q., Hoekstra, M., Kandasamy, P., Kelin, A. V., Milstein, S., et al. (2014). Multivalent N -acetylgalactosamine-conjugated siRNA localizes in hepatocytes and elicits robust RNAi-mediated gene silencing. J. Am. Chem. Soc. *136*, 16958–16961.

28. Nishina, K., Unno, T., Uno, Y., Kubodera, T., Kanouchi, T., Mizusawa, H., and Yokota, T. (2008). Efficient in vivo delivery of siRNA to the liver by conjugation of α-tocopherol. Mol. Ther. *16*, 734–740.

29. Tai, W. (2019). Current aspects of siRNA bioconjugate for in vitro and in vivo delivery. Molecules *24*, 2211.

30. Xia, C.-F., Boado, R.J., and Pardridge, W.M. (2009). Antibody-mediated targeting of siRNA via the human insulin receptor using avidin-biotin technology. Mol. Pharm. *6*, 747–751.

31. Zheng, Y.H., Jeang, K.T., and Tokunaga, K. (2012). Host restriction factors in retroviral infection: Promises in virus-host interaction. Retrovirology *9*, 112.

32. Hatziioannou, T., and Bieniasz, P.D. (2011). Antiretroviral restriction factors. Curr. Opin. Virol. *1*, 526–532.

33. Anderson, J.L., and Hope, T.J. (2005). Intracellular trafficking of retroviral vectors: obstacles and advances. Gene Ther. *12*, 1667–1678.

34. Galla, M., Schambach, A., Towers, G.J., and Baum, C. (2008). Cellular restriction of retrovirus particle-mediated mRNA transfer. J. Virol. *82*, 3069–3077.

35. Prel, A., Caval, V., Gayon, R., Ravassard, P., Duthoit, C., Payen, E., Maouche-Chretien, L., Creneguy, A., Nguyen, T.H., Martin, N., et al. (2015). Highly efficient in vitro and in vivo delivery of functional RNAs using new versatile MS2-chimeric retrovirus-like particles. Mol. Ther. Methods Clin. Dev. *2*, 15039.

36. Kasaraneni, N., Chamoun-Emanuelli, A.M., Wright, G.A., and Chen, Z. (2018). A simple strategy for retargeting lentiviral vectors to desired cell types via a disulfide-bond-forming protein-peptide pair. Sci. Rep. *8*, 10990.

37. Bender, R.R., Muth, A., Schneider, I.C., Friedel, T., Hartmann, J., Plückthun, A., Maisner, A., and Buchholz, C.J. (2016). Receptor-Targeted Nipah Virus Glycoproteins Improve Cell-Type Selective Gene Delivery and Reveal a Preference for Membrane-Proximal Cell Attachment. PLoS Pathog. *12*, e1005641.

38. Buchholz, C.J., Friedel, T., and Büning, H. (2015). Surface-Engineered Viral Vectors for Selective and Cell Type-Specific Gene Delivery. Trends Biotechnol. *33*, 777–790.

39. van der Loo, J.C.M., and Wright, J.F. (2016). Progress and challenges in viral vector manufacturing. Hum. Mol. Genet. *25*, R42–R52.

40. Shirley, J.L., de Jong, Y.P., Terhorst, C., and Herzog, R.W. (2020). Immune Responses to Viral Gene Therapy Vectors. Mol. Ther. *28*, 709–722.
